# Effects of Landscape Features on Wild Bee Assemblages in Agricultural Areas

**DOI:** 10.3390/insects17101054

**Published:** 2026-10-13

**Authors:** Jovana Raičević, Jovana Bila Dubaić, Aleksandar Ćetković, Milan Plećaš

**Affiliations:** Faculty of Biology, University of Belgrade, Studentski trg 16, 11000 Belgrade, Serbia; jovanabila@bio.bg.ac.rs (J.B.D.); acetkov@bio.bg.ac.rs (A.Ć.); mplecas@bio.bg.ac.rs (M.P.)

**Keywords:** wild bees, agricultural areas, landscape composition, bee functional traits

## Abstract

Agricultural intensification alters landscape functionality by reducing habitat heterogeneity and resource availability for wild bees. Despite extensive research on semi-natural habitats as important features for wild bees in agricultural landscapes, the role of other landscape components remains less commonly and consistently evaluated. We investigated how landscape composition and flowering plant richness relate to bee abundance, diversity, and functional-group composition across three spatial scales (500, 1000, and 1500 m) in an intensively managed agricultural landscape. Unexpectedly, semi-natural habitats showed limited effects, with responses restricted to specific metrics and spatial scales. The presence of rural settlements negatively affected total bee abundance, as well as female and male abundances. However, effects on total and female abundance disappeared after excluding *Apis mellifera* from analyses, indicating that managed honeybees strongly influenced community-level patterns. At the largest spatial scale, rural settlements positively affected *Andrena flavipes*. Roads and flowering plant richness influenced particular bee groups rather than showing general community patterns. The presence of water bodies positively affected bee abundance, while positive effects on diversity metrics became evident only after excluding *A. mellifera*. Our results demonstrate that responses of wild bee assemblages to landscape characteristics are highly context-dependent, varying among functional groups and spatial scales.

## Simple Summary

Agricultural intensification negatively affects wild bees by altering landscape functionality, thus reducing habitat diversity and resource availability. Semi-natural habitats are widely recognised as important for wild bees in agricultural landscapes, while the effects of other landscape elements have received less research attention. In this study, we examined how different landscape elements, including semi-natural habitats, rural settlements, roads, and water bodies, as well as local flowering plant richness, influence bee abundance and diversity in an intensively managed agricultural region. We found that landscape-level availability of semi-natural habitats had limited effects, while other landscape features variably affected specific bee groups. Water bodies were associated with higher bee abundance and diversity, whereas the effects of rural settlements differed among bee groups. We also found that the presence of managed honeybees can strongly influence overall patterns and may obscure responses of wild bees in analyses that do not discern between managed and wild bees. Responses of various bee groups depended on the spatial scale at which landscape features were evaluated. Our findings emphasise that understanding of wild bee communities requires consideration of the complexity of landscape features, the spatial scale of observation, and the varied ecological requirements of different bee groups.

## 1. Introduction

Pollinators are facing increasing pressure worldwide, with declines in abundance, diversity, and distribution linked to multiple interacting drivers, including habitat loss and fragmentation, agricultural intensification, pesticide use, changes in floral and nesting resources, invasive species, pathogens, and climate change [1,2,3]. Among these drivers, land-use change associated with agricultural expansion and intensification is considered one of the major pressures on bee communities, as it simplifies landscapes and reduces the availability, diversity, and continuity of key resources. This is particularly important because animal pollination contributes substantially to both crop production and the reproduction of wild plants, with approximately 85% of food crops benefiting to some degree from animal pollination and up to 90% of flowering plant species [2,4,5]. Insects, and particularly bees, are among the most important animal pollinators in both natural and agricultural systems.

In agricultural landscapes, semi-natural habitats are generally regarded as key habitats for wild bees because they can provide diverse foraging resources, nesting sites, shelter, and overwintering sites. They are highly variable in form, area, vegetation structure, floral richness, and management status, and include various grasslands, shrubs and woodlands, marginal herbaceous or woody elements (hedgerows, field and roadside verges, riparian habitats), and any other non-crop elements. These habitats are particularly important in intensively managed landscapes, where they can support bee abundance and diversity by increasing resource availability and landscape connectivity [6,7,8]. However, their value for bees depends not only on total cover, but also on habitat type, floral abundance, phenology, vegetation structure, and nesting-resource availability [9,10,11].

While conservation efforts often focus on improving specified semi-natural habitats to support wild bees in agricultural landscapes, agricultural mosaics also contain other non-crop landscape elements that may influence bee communities. More intensively used human-made landscape elements, such as rural settlements and roads, can strongly modify habitat heterogeneity, connectivity, resource availability, and disturbance levels, with varying effects on different wild bee populations. Rural settlements may contain gardens, orchards, yards, ruderal vegetation, ornamental plantings, but also buildings and other impervious structures, and their effects on bees may be highly context-dependent. In intensively managed farmlands, domestic gardens can support higher wild bee abundance and species richness in their immediate surroundings, suggesting that settlement-associated vegetation may act as an additional resource-providing element in agricultural landscapes [12]. However, studies comparing rural, suburban, and urban habitats also show that increasing urbanisation and impervious surface cover can alter bee community composition and reduce the occurrence of more sensitive functional groups, including solitary, soil-nesting, and oligolectic species [13,14,15]. Roads, including paved transport routes and unpaved field roads, may have both positive and negative effects on bee communities. Roadside vegetation can provide floral resources, nesting substrates, and movement corridors, particularly in intensively managed landscapes, while roads may also introduce disturbance through traffic, vegetation management, agrochemical exposure, and impacts on nesting substrates [16,17,18,19,20]. Their overall value for bees therefore depends strongly on local road and verge characteristics.

Water-associated habitats, such as drainage canals, ponds, wetlands, ditches, temporary water bodies, and their structurally and floristically variable riparian zones, represent another important group of non-crop elements in agricultural landscapes. They may be particularly relevant in highly simplified agricultural landscapes, where even small or thin and linear habitats can provide additional foraging or nesting opportunities. These water-associated habitats have received comparatively little attention in wild bee studies, since they are often included in a more generalised semi-natural vegetation category. However, available evidence suggests that ponds, temporary water bodies, ditches, and wetlands can support pollinator abundance, bee richness, and the availability of food and nesting resources [21,22,23]. Wet meadows and other wetland-associated habitats may also support specialised bee species linked to wetland plants [24]. By maintaining vegetation associated with higher water availability, these habitats may contribute to floral resource continuity in otherwise dry or intensively farmed landscapes, although this mechanism likely depends on local vegetation composition and hydrological conditions. In addition, reduced water availability and drought stress can negatively affect floral traits, flower production, pollen and nectar availability and quality, and plant-pollinator interactions, further suggesting that water-associated habitats may provide important complementary resources for bees during dry periods [25,26].

Local floral resources are also among the most important factors which shape bee communities. Flowering plant richness can increase the range of available pollen and nectar resources and is often positively related to bee abundance, species richness, and flower visitation [27,28,29]. However, flowering plant richness alone may not fully capture the functional value of floral resources for bees. Bee responses can also depend on floral abundance, resource density, flowering phenology, nectar and pollen quality, and the presence or dominance of particularly rewarding plant species. Thus, sites with similar flowering plant richness may differ substantially in their actual value for bees, while sites with relatively few flowering species may still provide abundant resources if dominated by dense stands of highly rewarding plants. Floral resources may also mediate interactions between managed honeybees (*Apis mellifera*) and wild bees, both through competition for shared pollen and nectar resources and through pathogen transmission, with honeybees potentially acting as reservoirs and vectors of pathogens at shared flowers [30,31].

In addition to community-level responses, bees may respond differently to landscape and local habitat features depending on their functional traits. Species differ in nesting strategy, sociality, diet breadth, body size, phenology, and mobility, and these traits can influence how they use nesting and foraging resources across simplified vs. heterogeneous landscapes. Habitat loss and landscape simplification can shift wild bee community composition because responses depend on dispersal capacity, diet breadth, and sociality [32]. These differences are also expected to be scale-dependent, because bees are central-place foragers that require nesting sites and floral resources within their foraging range to complete their life cycle. Since foraging range varies strongly among species and is closely related to body size and sociality, different bee groups may respond to landscape features at different spatial scales [33,34,35].

In this study, we examined how landscape composition and local flowering plant richness relate to bee communities in intensively managed agricultural landscapes of the south-eastern Pannonian Plain in Serbia. Specifically, we analysed the effects of semi-natural habitats, rural settlements, roads, and water bodies at three spatial scales, together with local flowering plant richness, on bee abundance, diversity, and functional-group composition. By considering both community-level responses and functional groups differing in nesting strategy, sociality, diet breadth, and body size, our aim was to assess whether different landscape and local habitat features are associated with distinct components of bee communities (assemblages) in a highly simplified agricultural mosaic.

Based on previous studies, we expected that landscape elements providing additional floral, nesting, or refuge resources, together with higher local flowering plant richness, would generally be associated with higher bee abundance and diversity. For more intensively used human-made elements, such as rural settlements and roads, we expected effects to be more variable because these features may provide additional resources but also introduce disturbance. Finally, we expected that responses would differ among functional groups and across spatial scales, reflecting differences in nesting strategy, diet breadth, body size, sociality, and foraging range.

## 2. Materials and Methods

### 2.1. Study Area and Bee Sampling

**Figure 1:**
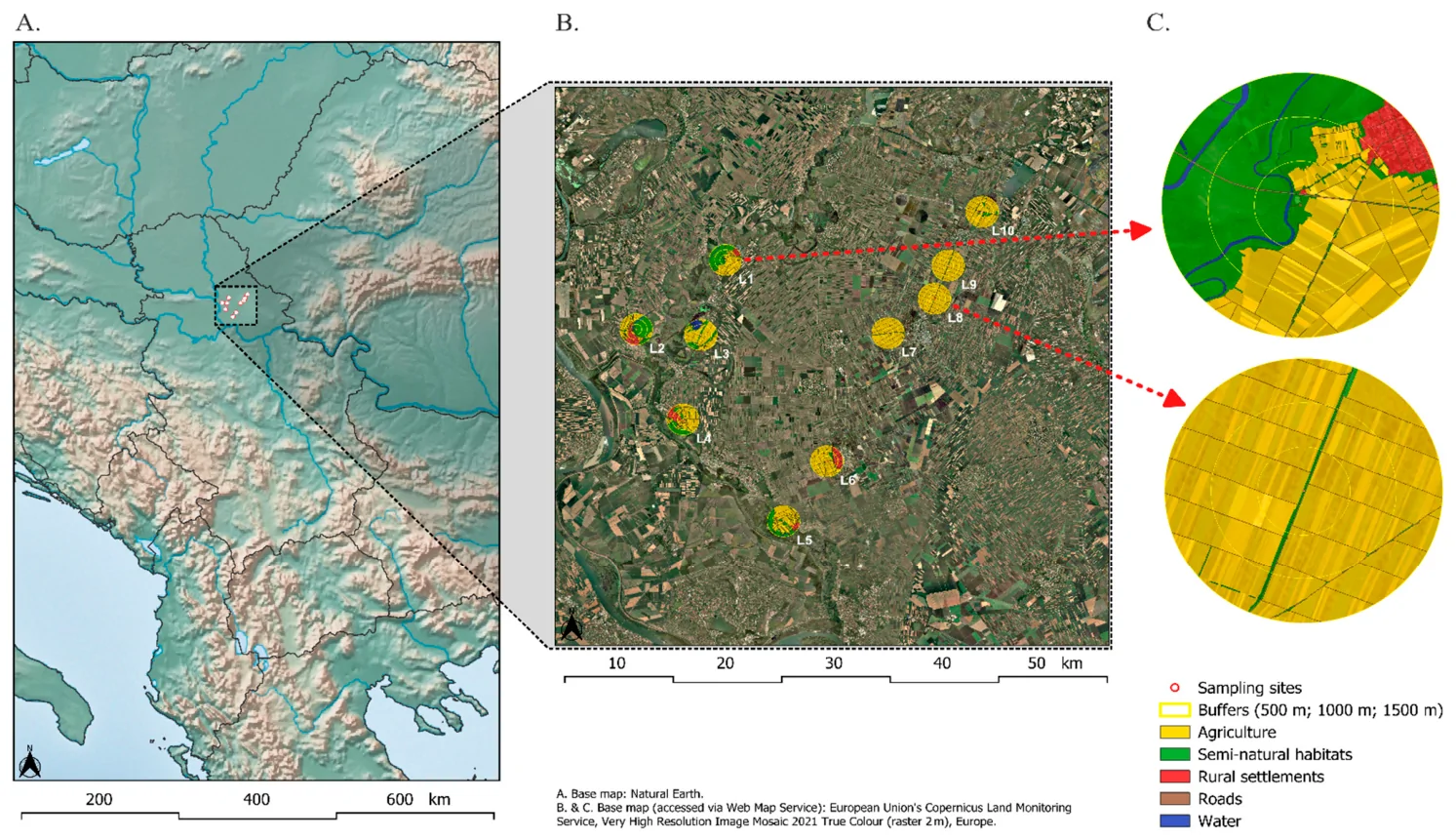
Study area and sampling sites. (**A**) Map of the Republic of Serbia with neighbouring countries; the inset square shows the sampled area within the South Banat subregion. (**B**) Satellite image showing spatial configuration of 10 sampled sites with digitised landscape features. (**C**) Detailed view of the landscape composition for sites L1 and L8 based on satellite imagery. Note: The background for all panels consists of publicly available datasets ((**A**) Natural Earth map; (**B**,**C**) European Union’s Copernicus Land Monitoring Service satellite image mosaic), while all landscape features were independently digitised by the authors.

The study was conducted in South Banat, a subregional unit of northern Serbia (Figure 1A) that forms the south-western part of the larger Banat area in the Pannonian Basin. South Banat is predominantly characterised by lowland terrain with a mean altitude of approximately 80 m a.s.l., and its landscape matrix is heavily shaped by intensive agriculture on fertile chernozem and alluvial soils [36]. Numerous meanders and oxbow lakes at various stages of terrestrialisation, along with an extensive network of drainage canals, are present across the subregion. Additionally, several small rural settlements (fewer than 5000 inhabitants each) are distributed across the area, interconnected by primary roads and numerous unpaved field roads. The climate is moderate continental, characterised by a mean annual temperature of approximately 11 °C and annual precipitation of about 600 mm that exhibits an uneven distribution throughout the year [37].

Biogeographically, the area belongs to the Pannonian biogeographical region and corresponds to the forest-steppe climatic domain, with associated steppe elements and locally developed azonal and intrazonal hygrophilic vegetation types [38,39]. However, the current land cover features greatly reduced and fragmented remnants of natural vegetation, due to intensive agriculture. Our study focused mainly on the remaining semi-natural habitats adjacent to arable fields, such as field margins, roadsides, and interstitial patches between fields. In this context, these linear fragments and rural settlement margins correspond to locations where remnants of altered steppe and ruderal vegetation persist. Strongly influenced by anthropogenic factors, these elements serve as critical ecological refuges that provide essential foraging and nesting resources for wild bees within the managed agricultural landscape.

We conducted wild bee surveys at 10 study sites in the South Banat region (Figure 1B). The distance between the nearest sites ranged from 3.5 km to 8.5 km, and the total area was approximately 35 × 35 km, with a geographical extent from 44.934081° to 45.243450° N latitude, and from 20.372336° to 20.819646° E longitude. Sampling was conducted twice during the 2020 field season, in June and early July, corresponding to the late spring and early summer period, when bee assemblages are typically well represented in the region, and the availability and diversity of floral resources are high. During each visit, we recorded all the plant taxa present at the site that were in flower, and we visually surveyed their attractiveness for bees. At each site, bee sampling was performed only on plant taxa which attracted a meaningfully representative bee assemblage, rather than just a few accidental visitors. Therefore, we either ignored or subsequently excluded plant taxa on which fewer than 10 bee individuals could be recorded. Due to practical difficulties in reliable field recognition of plant species, and also taking into account that foraging preferences of bee taxa are expressed and usually analysed at a coarser taxonomic level [40,41,42], our sampling was based on the plant genus level; effectively, many of the recorded genera were represented by only a single flowering species at each site. To ensure consistency, the same two researchers conducted all field estimates and sampling by working in a pair throughout the study (JR, MP). Each selected plant genus at a site was sampled for 15 min, and bees were collected with an entomological net if observed contacting the flowers. Only a few species that could be reliably identified in the field, primarily *A. mellifera* and some *Bombus* species, were not collected. The collected specimens were stored in vials with 70% ethanol and taken to the laboratory for further processing and identification. Most specimens were identified to species (by AĆ), except in a few problematic cases, as elaborated in Table S1 (see Remarks and references cited therein [43,44,45,46,47,48,49,50,51,52,53,54,55,56,57,58,59]).

The study sites were selected along a gradient of landscape complexity, defined by an increasing proportion of semi-natural habitats (1.9–45.7%) and a decreasing proportion of arable land (96.9–32.3%) (Table S2). Landscape composition was determined using QGIS (v. 3.38) [60] and the Copernicus Land Monitoring Service (CLMS) image mosaic [61], which was streamed via Web Map Service (WMS). Based on the satellite image mosaic, supplemented by field observations in a limited area, features were digitised within radii of 500 m, 1000 m, and 1500 m around the sampling sites and categorised into one of five predefined categories: agriculture, semi-natural habitats, rural settlements, roads, and water bodies (Figure 1C). The area and proportion of each category were calculated using QGIS geometry functions. Flowering plant richness at each site was calculated as the total number of plant genera flowering during the sampling period or, for the diversity analysis, as the total number of flowering plant genera per site (combined over two sampling periods).

### 2.2. Data Analyses

We analysed how bee abundance, bee diversity, and functional group abundance varied in relation to landscape and local variables. Bee abundance was analysed both as the total abundance of all bee species combined and as separate response variables, including total abundance excluding *A. mellifera*, total female abundance, female abundance excluding *A. mellifera*, and total male abundance, in order to disentangle group-specific responses. In addition, we analysed the total abundance of *A. flavipes* females, as the most abundant wild bee species. Functional groups were classified according to pollen foraging preference (oligolectic vs. polylectic), nesting behaviour (ground-nesting vs. cavity-nesting), social organisation (solitary vs. social), and body size (small, medium, and large), extracted from several literature sources [44,45,46,49,50,53,54,62,63,64,65]. Average body size values, expressed as body length (mm), were classified into three size categories (small: ≤7.5 mm; medium: >7.5 mm and ≤11.5 mm; large: >11.5 mm) according to [66]. Functional group analyses were based only on female individuals, after excluding parasitic species.

Landscape variables included the proportion of arable land, semi-natural habitats, rural settlements, roads, and the presence or absence of water bodies within the surrounding landscape. Flowering plant genera richness was included as the local variable: here we used the total number of plant genera that we recorded as flowering during each sampling event at each sampling site (this also included taxa which were not attractive to bees, hence not sampled). To assess scale-dependent responses, landscape composition was quantified within buffers of 500 m, 1000 m, and 1500 m around each sampling site. Spearman’s rank correlation analysis was performed to assess the relationship between four continuous landscape variables across all spatial scales, excluding water, which was treated as a binary (presence/absence) variable. Due to strong negative correlations between the proportions of arable land and semi-natural habitats across all spatial scales (*r_s_* ranged from −0.948 to −0.963, *p* < 0.001 for all scales; Figure S1), arable land was excluded from subsequent models.

We used generalised linear mixed models (GLMMs) to examine the relationships between bee abundance, functional group abundance, and landscape and local variables. As the number of samples varied across dates and sites due to differences in plant availability, we used the offset option with log-transformed total sampling duration to standardise all samples to the same effort, equivalent to one 15 min sampling. We used the Poisson family and the log-link function to account for the distribution of the data. Sampling date, nested within location, was included as a random effect to account for multiple sampling events at each location. Generalised linear mixed models were fitted using the R package lme4 (v. 1.1-35.5) [67].

To investigate bee diversity patterns, we standardised diversity estimates using coverage-based rarefaction and extrapolation implemented in the R package iNEXT (v. 3.0.1) [68]. Diversity was quantified using the estimateD function and expressed as Hill numbers of order *q* = 0 (species richness), *q* = 1 (Shannon diversity), and *q* = 2 (Simpson diversity) [69]. For the full dataset, estimates were standardised to a target sample coverage of 0.897 (Table S3), whereas for the dataset excluding *A. mellifera*, estimates were standardised to 0.855 (Table S4). These thresholds were selected to maximise comparability while minimising extrapolation beyond observed sample completeness. To examine relationships between diversity metrics and landscape and local variables, we fitted linear regression models (LMs) using log-transformed standardised Hill number estimates as response variables.

The variance inflation factor (VIF) was calculated for each model to test for collinearity between variables. Variables with a VIF value above 3 were sequentially excluded from the models. Model selection began with complete models containing all variables. Non-significant variables were removed sequentially, starting with the least significant predictor until further simplification did not improve model fit based on AIC comparison and residual diagnostics. Model validity was assessed by examining the residuals. The most appropriate landscape scale for each response variable was determined by the lowest AIC value. Residual spatial autocorrelation was assessed for each final model using Moran’s I on DHARMa residuals (DHARMa package v. 0.4.7) [70], with geographic distances between sampling sites calculated using the geosphere package (v. 1.6-8) [71]. No significant residual spatial autocorrelation was detected in any of the final models (Table S5).

The second sampling period at site L10 was omitted from all analyses because localised drought conditions induced premature senescence, leaving only one species in flower in insufficient numbers. All data analyses, forest plots, and Supplementary Figures were generated using R (v. 4.4.0) [72].

## 3. Results

Landscape composition varied substantially among study sites and spatial scales (Table S2). Arable land was generally the dominant land-cover type, although some sites (e.g., L1 and L2) contained relatively high proportions of semi-natural habitats. Water bodies and rural landscape elements were unevenly distributed across the study area, whereas road coverage remained consistently low. At the larger spatial scales (1000 and 1500 m), water bodies occurred at half of the sites, while at the smallest scale (500 m) they were recorded at only two sites. Rural settlements were absent from four sites across all spatial scales.

A total of 2088 specimens were collected, representing 96 species and morphospecies from 26 bee genera (Table S1). The most species-rich genera were *Lasioglossum* and *Eucera*, represented by 17 and 11 species, respectively, whereas 11 genera were represented by a single species only, including *Apis mellifera*. The honeybee was the most abundant species, accounting for 707 individuals, followed by *Andrena flavipes* with 374 individuals. Due to the high abundance of *A. flavipes*, *Andrena* was the most abundant wild bee genus, comprising more than a quarter of all collected wild bee specimens. Nearly one third of all recorded species (30) were represented by singletons. Females only were recorded for 55 species (nearly 60% of recorded species; 1200 specimens), males only for 11 species (13 specimens), while both sexes were recorded for 30 species (619 females and 256 males). For functional group analyses, *A. mellifera* was excluded as a managed species, as were parasitic bees, which comprised 12 species from five genera but only 16 specimens in total. The most abundant functional groups were ground-nesting (1056 specimens, 53 species), polylectic (869 specimens, 54 species), solitary (768 specimens, 55 species), and medium-sized bees (700 specimens, 28 species). Bee species richness per site ranged from 14 to 33 species, with 24.1 ± 7.0 (mean ± standard deviation) species per site (Table S1).

We analysed bee assemblages recorded on 21 plant genera from 8 plant families across all study sites, ranging from 2 to 7 genera per site (mean ± standard deviation: 4.1 ± 1.6). Most frequently sampled plants were *Melilotus* (seven sites), *Cirsium* (five sites), and *Thymus* (four sites), while the most diverse sampled plant families were Fabaceae (eight genera), Asteraceae (four genera) and Lamiaceae (three genera). The greatest number of bee species were recorded on *Cirsium* (31), *Mellilotus* (28), *Thymus* (20), *Anthemis* (19) and *Potentilla* (19). Highest bee abundances were recorded on *Mellilotus* (535), *Cirsium* (477), *Trifolium* (151) and *Astragalus* (118). Additionally, 19 plant genera were recorded in flowering, but either did not host any bee visitors during our survey or attracted a meaningless number of bees. Exclusion of such samples affected two plant genera and 10 individuals from the four most common bee taxa.

**Figure 2:**
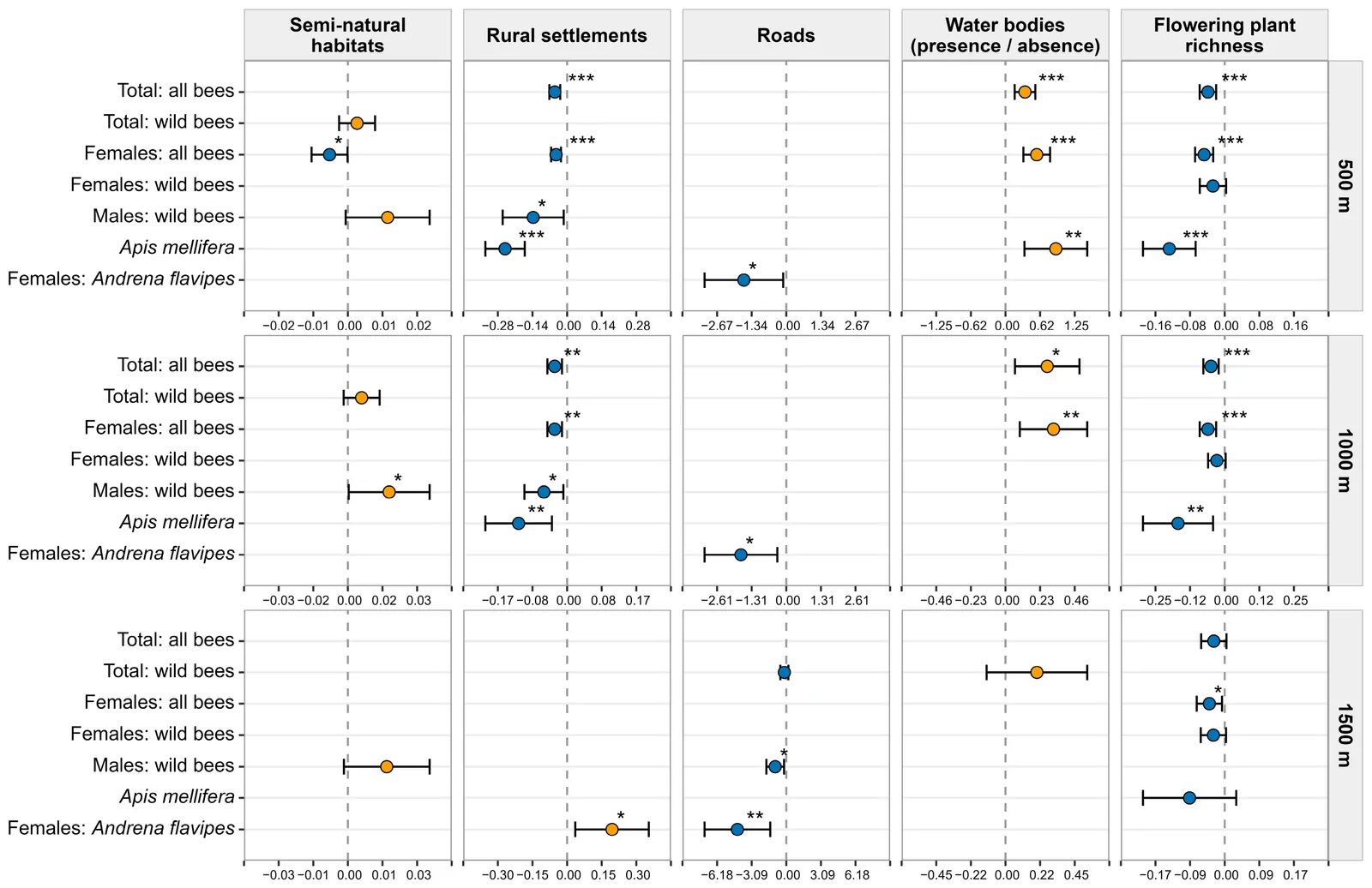
Forest plot of GLMM estimates for the effects of local and landscape variables across three spatial scales on the set of analysed bee abundance variables. Positive estimates are shown in orange and negative estimates in blue. Error bars indicate 95% confidence intervals (CI). Significance levels are indicated as *** *p* ≤ 0.001, ** *p* ≤ 0.01, * *p* ≤ 0.05. Final models are presented in Table S6.

The total abundance of bees responded negatively to the proportion of rural settlements and to flowering plant richness, while it responded positively to the presence of water bodies in the landscape at the 500 m and 1000 m scales. No significant effect was observed at the 1500 m scale. Female abundance showed a similar pattern, with an additional negative effect of the proportion of semi-natural habitats at the 500 m scale. However, after excluding *A. mellifera* from the total and female abundance datasets, none of the landscape or local variables had significant effects at any spatial scale. In contrast, when honeybee abundance was analysed separately, the proportion of rural settlements and flowering plant richness had a negative effect at both the 500 m and 1000 m scales, while the presence of water bodies had a positive effect, but only at the 500 m scale. Male abundance showed scale-dependent responses. At the 500 m and 1000 m scales, it responded negatively to the proportion of rural settlements, with an additional positive effect of semi-natural habitats at the 1000 m scale. However, at the 1500 m scale, male abundance showed a negative response to the proportion of roads. Female abundance of *A. flavipes* responded negatively to roads across all spatial scales, but positively to rural settlements at the 1500 m scale (Figure 2; Table S6).

**Figure 3:**
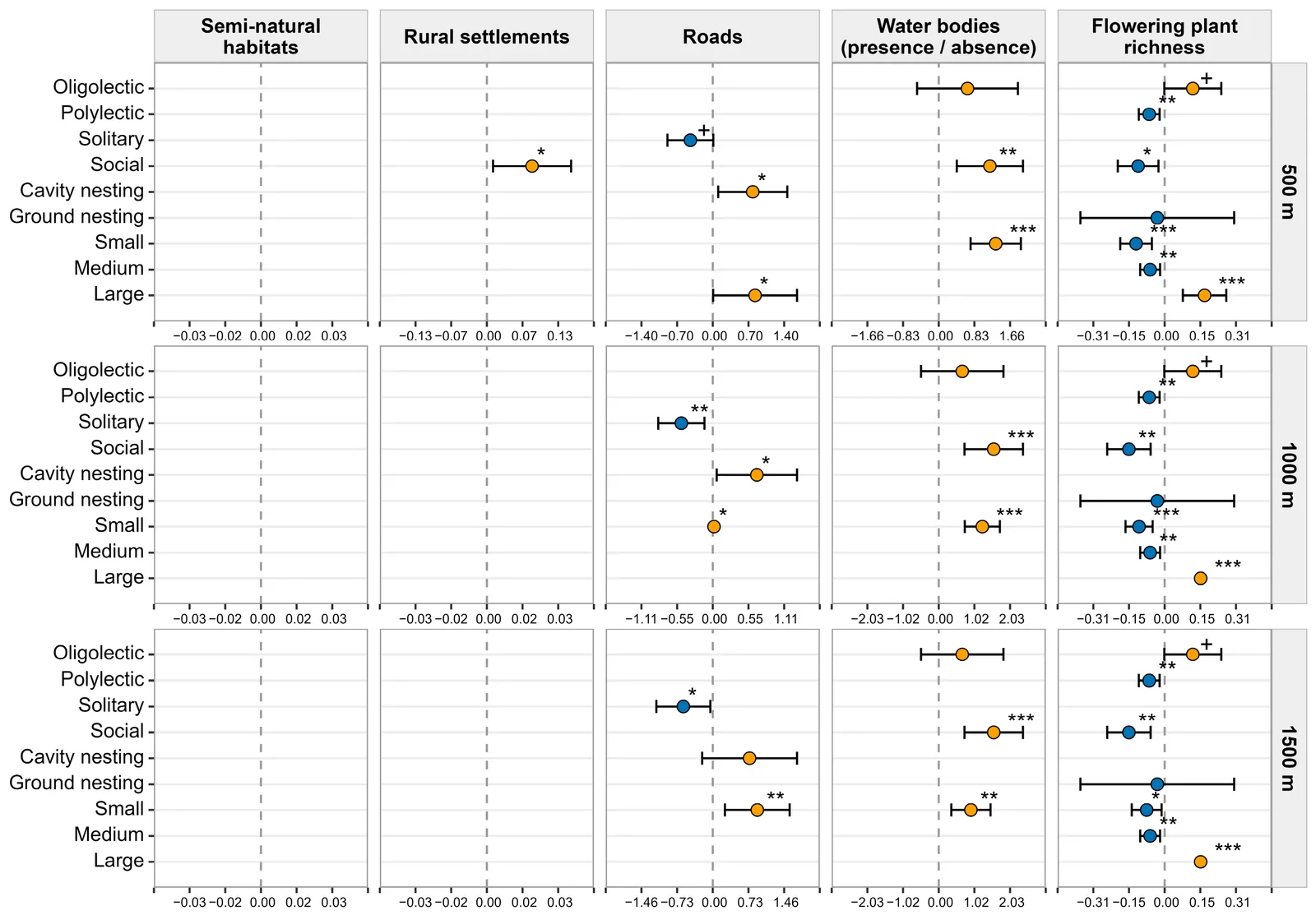
Forest plot of GLMM estimates for the effects of local and landscape variables across three spatial scales on the set of analysed functional group abundance variables. Positive estimates are shown in orange and negative estimates in blue. Error bars indicate 95% confidence intervals (CI). Significance levels are indicated as *** *p* ≤ 0.001, ** *p* ≤ 0.01, * *p* ≤ 0.05; “+” indicates marginally significant effects (0.05 < *p* < 0.06). Final models are presented in Table S6.

Neither oligolectic nor polylectic species showed significant responses to landscape variables at any spatial scale. However, flowering plant richness had a marginally significant positive effect on oligolectic species (*p* = 0.053) and a significant negative effect on polylectic species. Social species responded positively to the presence of water bodies and negatively to flowering plant richness across all spatial scales, while the proportion of rural settlements had an additional positive effect at the 500 m scale. In contrast, solitary species were negatively affected only by road coverage, although this effect was marginally significant at the 500 m scale (*p* = 0.056). Cavity-nesting bees were positively affected by the proportion of roads at the 500 m and 1000 m scales, whereas no significant effects were detected for ground-nesting bees. Small species responded positively to water bodies and negatively to flowering plant richness across all scales, with road coverage having an additional positive effect at the two larger scales. Medium-sized species were negatively affected only by flowering plant richness, while large-bodied species showed the opposite pattern, responding positively to flowering plant richness, with an additional positive effect of roads at the 500 m scale (Figure 3; Table S6).

**Figure 4:**
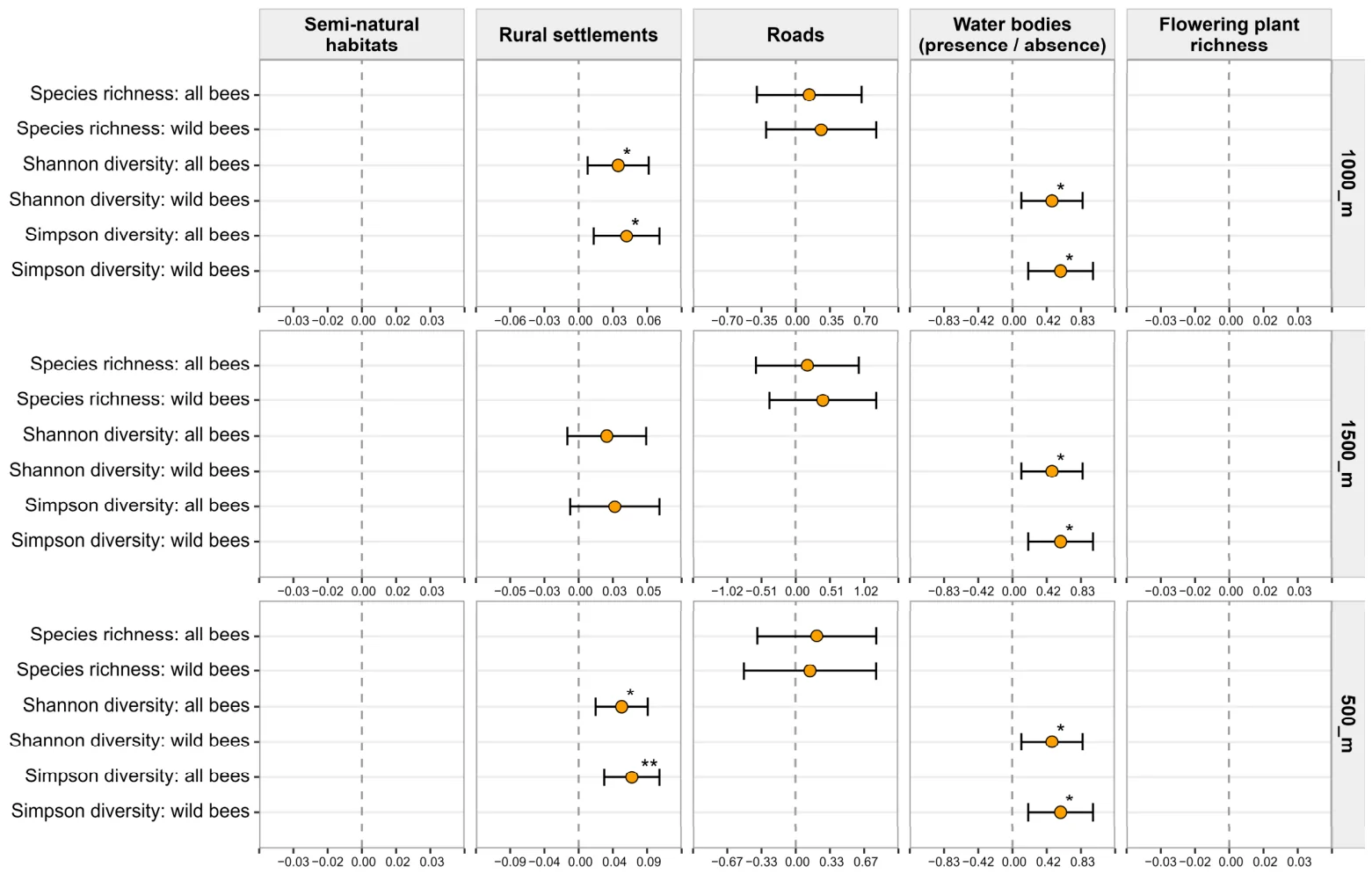
Forest plot of LM estimates for the effects of local and landscape variables across three spatial scales on the set of analysed diversity variables. Positive estimates are shown in orange. Error bars indicate 95% confidence intervals (CI). Significance levels are indicated as, ** *p* ≤ 0.01, * *p* ≤ 0.05. Final models are presented in Table S7.

Species richness was not affected by any landscape or local variables, regardless of whether *A. mellifera* was included in the analyses. Shannon and Simpson diversity showed similar responses when honeybees were included, with the proportion of rural settlements having a positive effect at the 500 m and 1000 m scales, but not at the 1500 m scale. In contrast, after excluding *A. mellifera*, only the presence of water bodies showed a positive effect across all spatial scales (Figure 4; Table S7).

For most response variables influenced by landscape factors, including total bee abundance, female abundance, male abundance, honeybee abundance, social species, large species, and Shannon and Simpson diversity, models at the 500 m scale provided the best fit. In contrast, models for *A. flavipes* female abundance and cavity-nesting species were best supported at the 1000 m scale, while models for small species performed best at the 1500 m scale.

## 4. Discussion

Our study explores the complex relationships between landscape features and wild bee communities, focusing on the proportion of semi-natural habitats, rural settlements, and roads and the presence of water bodies within the landscapes, as well as flowering plant richness within the sampled semi-natural fragments. We found that bee abundance, diversity and functional traits responded in a complex and scale-dependent way to landscape and local variables measured at three spatial scales (500, 1000, and 1500 m radius). Rather than showing uniform responses across the bee community, individual predictors affected only particular abundance, diversity, or functional-group metrics, while some variables showed no detectable response. These findings underline the context-specific nature of bee–habitat relationships and highlight the importance of distinguishing among response variables, functional groups, and managed versus wild bees.

### 4.1. Semi-Natural Habitats

Semi-natural habitats are generally expected to support wild bee communities in agricultural landscapes, as they provide floral resources, nesting sites, shelter, and overwintering habitats, as shown by numerous studies [6,7,8,9,10]. In our study, however, this pattern was not consistently observed. For most response variables, the proportion of semi-natural habitats had no significant effect, while a negative effect on female abundance and a positive effect on male abundance were detected only at single spatial scales. Given the strong negative correlation between the proportion of arable land and semi-natural habitats, the observed lack of a relationship with semi-natural habitats may also suggest a similarly weak relationship with arable land. Likewise, the negative relationship between semi-natural habitats and female abundance, and the positive relationship with male abundance, would suggest relationships in the opposite direction with arable land, although these complementary patterns should be interpreted cautiously because only the proportion of semi-natural habitats was included directly in the models.

Within the range of landscape conditions covered by our study, the amount of semi-natural habitat alone did not appear to be a limiting factor for most bee response variables. This suggests that the quantity of semi-natural habitats alone may not adequately represent their functional quality and may therefore not always be a good metric of habitat value for bees. One possible explanation is that semi-natural habitats in our landscapes differed in functional quality. Semi-natural habitats can vary substantially in their value for bees depending on habitat type, vegetation structure, floral abundance, phenology, and availability of nesting resources. Recent studies have shown that different types of semi-natural habitats support different wild bee assemblages and provide complementary foraging and nesting resources, suggesting that combining them into a single broad category can obscure habitat-specific effects [9,10,11]. Therefore, a high proportion of semi-natural habitat does not necessarily imply high-quality bee habitat if these areas are florally poor, heavily disturbed, or unsuitable for nesting. This may be particularly relevant in our study area, where the landscapes with the highest proportions of semi-natural habitats were dominated mainly by occasionally flooded grasslands and intensively grazed pastures. Although these habitat types may contribute to landscape heterogeneity and the overall amount of non-crop vegetation, their value for bees may be limited if flooding, intensive grazing or seasonal vegetation changes reduce floral abundance and continuity, or if suitable nesting substrates are scarce. Thus, landscapes with a relatively high proportion of semi-natural habitat may still provide only a limited amount of functionally suitable habitat for bees. In this sense, habitat quality and resource continuity may be more important than total semi-natural habitat cover alone. Similarly, arable land itself is not a uniform habitat category, and differences in crop type and management may also influence its value or impact on bees. Within the wider study area, the arable land is heavily dominated by cereals (63.8%), followed by sunflower (20.6%), soybean (8.1%), and oilseed rape (2.7%), with various other crops occupying the remaining 4.8% [73]. Sunflower and oilseed rape can provide substantial nectar and pollen resources during flowering and are commonly visited by managed honeybees. Their flowering periods, however, differed in relation to our sampling period. Oilseed rape flowering generally ends by May and therefore preceded our sampling, whereas sunflower flowering typically begins in early July, with timing varying according to sowing date and cultivar. Our sampling was conducted mainly in June, with a smaller proportion in early July, when sunflower flowering may have only partially commenced. In contrast to female bees, as males do not collect pollen for brood provisioning and are not constrained by nesting-site requirements, their response to different arable or semi-natural habitats may differ from that of females. However, because male abundance was relatively low compared to female abundance, and because we did not separately quantify different arable and semi-natural habitat types, these explanations should be considered tentative.

### 4.2. Rural Settlements

While several studies suggest that rural areas along an urbanisation gradient, from rural to urban environments, may support greater abundance and richness of wild bees [13,74,75,76,77,78,79], there is a lack of research specifically examining the impact of rural settlements within agricultural landscapes. Although rural and urban areas are typically mapped and quantified in landscape studies, they are often not analysed as independent predictors. Instead, they are usually incorporated indirectly through broader measures of landscape heterogeneity (e.g., Shannon landscape diversity index) or are treated mainly as residual land-cover categories relative to other more commonly analysed categories such as agricultural and semi-natural habitats.

In our study, we observed a negative effect of rural settlements on total bee abundance, affecting both female and male bees separately, as well as the abundance of *A. mellifera* when analysed independently. However, when honeybee abundance was excluded from total and female bee abundance, these effects disappeared. This suggests that the observed response of the entire bee assemblage was largely driven by the numerical dominance of honeybees, and that rural settlements may have little direct influence on wild bee abundance within agricultural landscapes. Because *A. mellifera* is a managed species whose distribution and abundance are strongly influenced by human activities, a negative relationship may reflect beekeeping practices rather than ecological responses of wild bees. In intensive agricultural landscapes, managed hives are often seasonally moved and concentrated near mass-flowering crops for pollination and honey production, which could explain lower abundance of honeybees in landscapes with a higher proportion of rural settlements during these periods. In contrast, rural settlements may provide various ‘green’ habitat elements potentially beneficial for wild bees, including yards, orchards, small gardens, and ornamental flower beds. However, these areas are also characterised by frequent human disturbance and vegetation management, which may contribute to the relatively weak overall response observed in wild bees.

Interestingly, we found a positive effect of rural settlements on the abundance of female *A. flavipes*. As one of the most common *Andrena* species, *A. flavipes* is a bivoltine, polylectic, solitary, ground-nesting bee that often forms aggregations of various sizes [80]. Although urbanisation is generally considered detrimental to ground-nesting species due to increasing impervious surfaces [81], rural settlements typically contain a greater proportion of permeable and vegetated surfaces, which may provide favourable nesting and foraging conditions for at least some ground-nesting bees within agricultural landscapes. We also found that social bees responded positively to the presence of rural settlements. Because social species maintain active colonies over a longer part of the season, they require continuous floral resources for colony development. In this context, rural settlements and the associated diversity of ‘green’ habitat types may increase both the temporal continuity and heterogeneity of resources available within agricultural landscapes. In addition, rural settlements positively influence bee diversity. The variety of ‘green’ habitat types associated with rural settlements may provide numerous foraging and nesting opportunities, thereby contributing to higher bee diversity within surrounding agricultural landscapes, particularly at small to medium spatial scales.

### 4.3. The Presence of Water Bodies

Interestingly, the presence of water bodies in the landscape emerged as a positive factor for both total bee abundance and female abundance. However, as observed with rural settlements, this effect disappeared after excluding *A. mellifera* from the analyses, again suggesting that overall abundance patterns were strongly influenced by honeybee dominance. In contrast, the positive effect of water bodies on diversity metrics became apparent only after honeybees were excluded.

In addition, both social bees and small species responded positively to the presence of water bodies in the surrounding landscape. Water-associated landscape elements such as canals, drainage ditches, riparian vegetation, and moist field margins may increase habitat heterogeneity within otherwise homogeneous agricultural landscapes. Such habitats can also provide more stable microclimatic conditions and prolonged flowering periods during summer, potentially supporting greater continuity of floral resources. Previous studies have shown that riparian and wetland habitats often support high floral abundance and pollinator activity [22,82,83,84], while soil moisture prevents drought stress and the associated reduction in flower production and nectar availability in many plant species [25]. In highly intensified agricultural landscapes, these wetland linear elements may therefore function as important resource corridors and refuges for wild bees.

### 4.4. Roads

Roads are often treated ambiguously in pollinator studies because they may function both as disturbance elements and as favourable linear habitats functioning as corridors [17,20]. Roads can negatively affect pollinators through traffic mortality, pollution, mowing, pesticide or herbicide exposure, and barrier effects. On the other hand, road verges may provide floral resources, nesting substrates, and corridors within otherwise simplified agricultural landscapes. In some cases, road-facing hedgerow margins and verges can support higher floral abundance, flower species richness, and bumblebee abundance than adjacent crop-facing margins [16,17]. Other roadside habitats have also been shown to support high flower and pollinator abundance, particularly in intensively managed landscapes, although their value depends strongly on habitat width, traffic intensity, mowing regime, and local management [18,19].

In our study, road cover did not have a detectable effect on total bee abundance or total female abundance, indicating that roads did not have a clear impact on the bee community as a whole. Instead, their effects appeared to be specific to certain groups. The most evident negative response was observed in *A. flavipes* (when only females are considered), which declined with increasing road cover across all spatial scales. As the most abundant wild bee species in our study, *A. flavipes* likely contributed substantially to the similar pattern observed for solitary bees as a group. In our study area, unpaved field roads accounted for approximately two-thirds of total road cover at all three spatial scales. Given their predominance, the observed negative relationship with total road cover may therefore be more strongly related to unpaved field roads than to paved roads. These roads may be associated with repeated passage of agricultural machinery, causing soil disturbance and compaction, as well as dust deposition and exposure to agrochemical drift from adjacent fields, potentially reducing their suitability for ground-nesting species such as *A. flavipes*. The strength of these effects may also depend on road-use intensity and local soil conditions. The absence of a similarly negative response for ground-nesting bees as a whole may reflect the ecological heterogeneity of this group, as ground-nesting species differ in substrate requirements and their ability to use sparsely vegetated or compacted soils [85].

In contrast, cavity-nesting bees showed a positive response to road cover. This effect is unlikely to be related to the road surface itself, but rather to roadside features such as hedgerows, shrubs, dry herbaceous stems, wooden fences, and abandoned structures that may provide better nesting opportunities in open agricultural landscapes [86]. Similar mechanisms may partly explain the positive responses observed for small and large groups, although these patterns are more difficult to interpret because body size encompasses several ecological traits, including mobility, foraging range, and resource use.

### 4.5. Flowering Plant Richness

Flowering plant richness is generally expected to benefit bee communities by increasing the diversity of available pollen and nectar resources. Many studies have shown positive effects of higher floral richness on pollinator abundance and diversity [27,28,29,87,88,89]. In our study, local flowering plant richness had a negative effect on total bee abundance and female abundance, which seems counterintuitive. However, these effects disappeared after *A. mellifera* was excluded from the analyses. Similar to the effects observed for some landscape variables, this suggests that the negative response was largely driven by honeybee abundance, rather than by wild bee assemblages. Although higher floral diversity is often linked to greater bee abundance and diversity [7,29], this relationship was not clearly reflected in our study.

This lack of a clear response of wild bees may indicate that flowering plant richness was not a limiting factor across the sampled sites. This result may reflect the fact that different sites could have provided favourable foraging conditions in different ways. Sites with higher flowering plant richness may have offered a broader range of pollen and nectar resources, whereas sites with fewer flowering plant genera may have been dominated by dense stands of highly rewarding plants. Therefore, both floristically rich sites and less diverse but resource-abundant sites may have provided sufficient foraging opportunities for wild bees. Moreover, bee sampling was conducted only on the fraction of plant genera present at each site, those which were sufficiently attractive for bees at the time of sampling, whereas our variable “flowering plant richness” included all the plant genera in flowering. As a result, at floristically less diverse sites, sampling probably covered a large proportion of the available flowering resources, while at floristically rich sites some less attractive flowering species were not visited, and consequently, not sampled. Therefore, our sampling approach and the logistic constraints limit our ability to separate the effects of flowering plant richness from other relevant aspects of floral resource availability.

The negative response of social bees may reflect their dependence on abundant and predictable floral resources. Social bees maintain active colonies for a longer part of the season and require a continuous supply of pollen and nectar for colony development; therefore, they may respond more strongly to the quantity, continuity, and quality of floral resources than to the number of flowering plant genera alone. This interpretation is supported by studies showing that bumblebee colony performance can depend on floral resource continuity and local flower dominance rather than floral richness [90,91].

Similarly, polylectic species can exploit a wide range of plant taxa but may still concentrate on locally abundant and highly rewarding floral resources when these are available. Thus, sites with fewer flowering plant genera but dense stands of resource-rich plants may provide more efficient foraging conditions for many polylectic species than floristically richer sites with more dispersed resources. Previous studies have shown that floristic richness does not always translate into greater foraging activity, and that low-diversity patches or even monocultures can attract high numbers of bees, particularly social bees such as honeybees and bumblebees [92]. This may occur because highly diverse floral assemblages can increase search and handling costs, whereas dense patches of resource-rich flowers allow bees to optimise foraging efficiency [92,93,94,95]. In contrast, as oligolectic bees depend on the presence of specific suitable host plants, higher flowering plant richness may therefore increase the probability that appropriate hosts are present [96,97]. On the other hand, the negative effect observed for *A. mellifera* may reflect its tendency to exploit dense, resource-rich floral patches rather than species-rich but less concentrated floral assemblages. In addition, honeybee abundance may be influenced by management-related factors, such as the seasonal placement of hives near mass-flowering crops or other highly rewarding floral resources.

Because bee foraging range generally increases with body size, small and medium bees might be expected to respond more strongly to local floral conditions than large bees [31,98]. However, our results showed the opposite pattern: small and medium bees responded negatively to flowering plant richness, whereas large bees responded positively. This suggests that flowering plant richness alone may not reflect the availability of resources relevant to smaller bees. Smaller species may depend more strongly on nearby, spatially concentrated and easily exploitable floral resources, while large-bodied bees, due to their greater mobility, may be better able to use more dispersed resources across floristically richer sites.

### 4.6. Limitations

The interpretation of our results should take into account the following methodological considerations. The study was conducted across 10 independent landscapes during a single field season (2020), with both sampling periods restricted to early summer. Although this period generally coincides with high bee activity and floral resource availability in the region, the sampling window was constrained by COVID-19 restrictions and therefore did not capture seasonal variation in bee assemblages and floral resources.

Second, different semi-natural and arable habitat types were grouped into broader categories. Habitat classification was based on satellite imagery, as time and logistical constraints prevented detailed field surveys of all habitat patches. This allowed us to quantify habitat cover but not to assess habitat quality in detail or reliably distinguish among finer habitat types.

Finally, paved and unpaved roads were combined into a single road-cover variable. Given the limited number of independent landscapes (*n* = 10) and low variation in each road type, particularly paved roads, their individual effects could not be reliably estimated. Modelling them separately would have increased model complexity relative to the information available for estimating their independent effects. As unpaved field roads accounted for approximately two-thirds of total road cover across all spatial scales, observed relationships with total road cover may predominantly reflect variation in this road type.

## 5. Conclusions

The results of our study revealed that associations between landscape features and bee assemblages varied depending on bee group and spatial scale in our landscapes strongly dominated by agriculture. Importantly, several apparent relationships involving total bee abundance were driven by the high abundance of the managed honeybee, *Apis mellifera*. When *A. mellifera* was excluded, these relationships changed, indicating that the presence of this managed species can influence patterns observed for the overall bee assemblages and obscure responses of wild bees. This highlights an important methodological consideration: managed *A. mellifera* and wild bees should be evaluated separately when examining relationships between landscape characteristics and bee communities, particularly in highly agricultural landscapes where the local abundance of *A. mellifera* may be strongly influenced by beekeeping practices. Additionally, the spatial extent of landscape features alone may not fully capture their influence on wild bee responses, highlighting the importance of considering the quality, availability, and functional role of the habitats and resources they provide. Assessing both the extent and characteristics of landscape features may therefore provide a more accurate understanding of their relationships with wild bee communities. However, our interpretation is constrained by the single-year study period and early-summer sampling window, as well as by the broad classification of semi-natural habitats due to limitations in field-based habitat assessment.

Future research building on these findings should test the consistency of the observed relationships across multiple seasons and years and under different agricultural conditions. Extending the sampling period and spatial scope, while incorporating more detailed field-based assessments of habitat quality and maintaining separate analyses of managed *A. mellifera* and wild bees, would help determine whether the patterns observed here persist across different temporal and environmental conditions and provide stronger evidence of the factors shaping wild bee responses to landscape characteristics in highly agricultural areas.

## Data Availability

The original contributions presented in this study are included in the article/Supplementary Material. Further inquiries can be directed to the corresponding author.

## Supplementary Materials

The following supporting information can be downloaded at: https://www.mdpi.com/article/10.3390/insects17101054/s1, Figure S1. Correlation matrices of landscape variables calculated at three spatial scales: (A) 500 m, (B) 1000 m, and (C) 1500 m. Diagonal panels show variable distributions, lower panels show pairwise relationships, and upper panels display Spearman’s rank correlation coefficients; Table S1. Taxonomic classification, functional traits, and abundance of collected species across study sites and plant genera (followed by Remarks on taxonomic issues concerning selected taxa in the dataset (identity, recognition, faunistics); Table S2. Geographical coordinates of the ten study sites and their landscape composition across three spatial scales; Table S3. Compiled iNEXT output of observed characteristics and estimated diversity metrics for the full dataset, standardized to a fixed sample coverage of 89.7% (estimateD function); Table S4. Compiled iNEXT output of observed characteristics and estimated diversity metrics for the dataset with *Apis mellifera* excluded, standardized to a fixed sample coverage of 85.5% (estimateD function); Table S5. Results of Moran’s I test for spatial autocorrelation across three spatial scales; Table S6. Results of generalized linear mixed models (GLMMs) examining the effects of four landscape variables and one local variable (flowering species richness) on bee abundance across three spatial scales (A = 500 m; B = 1000 m; C = 1500 m), including total bee abundance, bee subsets, and functional group abundances; Table S7. Results of linear regression models (LMs) examining the effects of four landscape variables and one local variable (flowering species richness) on bee diversity metrics across three spatial scales.

## Abbreviations

The following abbreviations are used in this manuscript:

AIC	Akaike Information Criterion
CI	Confidence Intervals
CLMS	Copernicus Land Monitoring Service
GLMMs	Generalised Linear Mixed Models
LMs	Linear Regression Models
VIF	Variance Inflation Factor
WMS	Web Map Services
