# Supplementary material for: Effects of Landscape Features on Wild Bee Assemblages in Agricultural Areas
Source: Insects. 2026 Oct 13;17(10):1054. doi: 10.3390/insects17101054 (PMC13704973; doi:10.3390/insects17101054)
Supplement: Supplementary file 1 [file insects-17-01054-s001.zip › insects-4547919-supplementary.pdf]

Figure S1. Correlation matrices of landscape variables calculated at three spatial scales: (A) 500 m, (B) 1000 m, and (C) 1500 m. Diagonal panels show variable distributions, lower panels show pairwise relationships, and upper panels display Spearman's rank correlation coefficients.

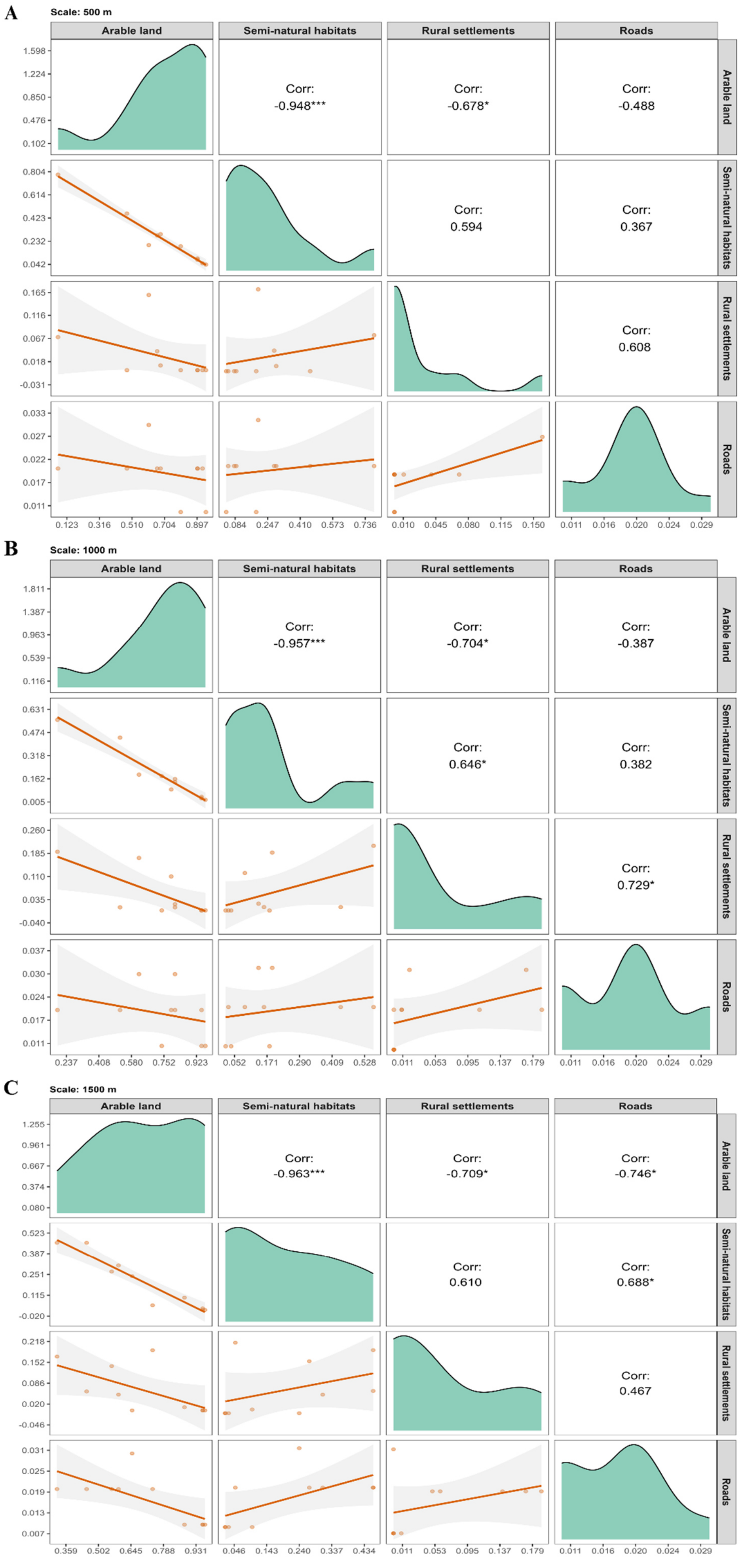

Table S1. Taxonomic classification, functional traits, and abundance of collected species across study sites and plant genera.

Taxa	Traits				Abundance per site										Abundance per plant																				
	Nesting	Pollen preference	Sociality	Size	L1	L2	L3	L4	L5	L6	L7	L8	L9	L10	*Achillea*	*Anchusa*	*Anthemis*	*Astragalus*	*Centaurea*	*Cirsium*	*Convolvulus*	*Coronilla*	*Delphinium*	*Lotus*	*Glechoma*	*Knautia*	*Lathyrus*	*Medicago*	*Melilotus*	*Potentilla*	*Ranunculus*	*Salvia*	*Thymus*	*Trifolium*	*Vicia*
Colletidae																																			
Colletinae																																			
1 *Colletes daviesanus*	Ground	Oligolectic	Solitary	Medium	-	1	-	3	-	-	-	-	-	-	1	-	3	-	-	-	-	-	-	-	-	-	-	-	-	-	-	-	-	-	-
2 *Colletes mlokossewiczi* ˣ ▪	Ground	Oligolectic	Solitary	Medium	-	1	-	-	-	-	-	-	-	-	1	-	-	-	-	-	-	-	-	-	-	-	-	-	-	-	-	-	-	-	-
3 *Colletes similis*	Ground	Oligolectic	Solitary	Medium	-	11	-	12	-	-	-	-	-	7	11	-	12	-	-	-	-	-	-	-	-	-	-	-	-	-	-	1	6	-	-
Hylaeinae																																			
4 *Hylaeus angustatus*	Cavity	Polylectic	Solitary	Small	-	-	-	-	-	-	3	-	-	1	-	3	-	-	-	-	-	-	-	-	-	-	-	-	-	-	-	1	-	-	-
5 *Hylaeus cornutus*	Cavity	Polylectic	Solitary	Small	-	-	-	1	-	-	-	-	-	-	-	-	1	-	-	-	-	-	-	-	-	-	-	-	-	-	-	-	-	-	-
6 *Hylaeus dilatatus*	Cavity	Polylectic	Solitary	Small	1	-	-	-	-	-	-	-	-	-	1	-	-	-	-	-	-	-	-	-	-	-	-	-	-	-	-	-	-	-	-
7 *Hylaeus imparilis*	Cavity	Polylectic	Solitary	Small	1	1	-	-	-	-	-	-	-	-	1	-	-	-	-	-	-	-	-	-	-	-	-	-	-	1	-	-	-	-	-
***8** *Hylaeus (Lambdopsis)* sp.	Cavity	Polylectic	Solitary	Small	-	1	-	-	-	-	-	-	-	-	-	-	-	-	-	-	-	-	-	-	-	-	-	-	-	1	-	-	-	-	-
Andrenidae																																			
Andreninae																																			
***9** *Andrena* cf. *afzeliella*	Ground	Polylectic	Solitary	Medium	10	7	3	-	-	4	1	8	-	-	-	-	-	-	-	-	-	-	-	-	-	-	-	-	32	-	-	-	-	1	-
10 *Andrena dorsata*	Ground	Polylectic	Solitary	Medium	3	-	-	-	-	1	-	-	-	-	-	-	-	-	-	-	-	-	-	-	-	-	-	-	4	-	-	-	-	-	-
11 *Andrena flavipes*	Ground	Polylectic	Solitary	Medium	100	39	40	3	-	51	55	74	12	-	21	-	3	-	-	14	-	-	-	-	-	-	-	-	327	1	1	-	6	1	-
12 *Andrena hattorfiana*	Ground	Oligolectic	Solitary	Large	-	-	-	-	2	-	-	-	-	-	-	-	-	-	-	-	-	-	-	-	-	2	-	-	-	-	-	-	-	-	-
13 *Andrena labialis*	Ground	Oligolectic	Solitary	Large	18	-	1	-	-	1	-	-	-	-	-	-	-	-	-	-	-	-	-	-	-	-	4	-	8	-	-	6	2	-	-
14 *Andrena labiata* ˣ ▪	Ground	Polylectic	Solitary	Medium	-	1	-	-	-	-	-	-	-	-	-	-	-	-	-	-	-	-	-	-	-	-	-	-	-	1	-	-	-	-	-
15 *Andrena rosae* ˣ ▪	Ground	Polylectic	Solitary	Large	1	-	-	-	-	-	-	-	-	-	-	-	-	-	-	-	-	-	-	-	-	-	-	-	1	-	-	-	-	-	-
16 *Andrena variabilis*	Ground	Polylectic	Solitary	Large	21	10	1	-	-	-	-	2	-	2	-	-	-	-	-	-	-	-	-	-	-	-	-	-	32	-	-	-	4	-	-
Halictidae																																			
Halictinae																																			
17 *Halictus asperulus*	Ground	Polylectic	Social	Medium	-	6	-	-	-	-	-	-	-	1	-	-	-	-	-	-	-	-	-	-	-	-	-	-	1	5	-	-	1	-	-
***18-19** *Halictus brunnescens / quadricinctus*	Ground	Polylectic	Solitary	Large	2	-	-	-	-	1	-	-	2	4	-	-	-	-	-	1	-	-	-	-	-	-	-	-	-	-	-	3	5	-	-
20 *Halictus maculatus*	Ground	Polylectic	Social	Medium	15	6	5	10	4	1	-	-	-	6	9	-	10	-	-	10	1	-	-	-	-	-	1	-	9	2	1	-	4	-	-
21 *Halictus patellatus*	Ground	Polylectic	Social	Large	1	-	3	1	1	-	-	3	-	2	-	-	1	1	-	3	1	-	-	-	-	-	-	-	3	-	-	-	2	-	-
22 *Halictus sexcinctus*	Ground	Polylectic	Solitary	Large	-	-	1	-	-	-	-	-	-	3	-	-	-	-	-	1	-	-	-	-	-	-	-	-	-	-	-	1	2	-	-
***23** *Halictus (Monilapis)* spp.	Ground	Polylectic	Social	Medium	3	24	15	23	17	6	1	-	1	14	6	1	23	1	-	19	1	-	-	-	-	9	2	-	4	13	5	6	9	4	1
24 *Lasioglossum calceatum*	Ground	Polylectic	Social	Medium	1	-	-	-	2	-	-	-	-	-	1	-	-	-	-	-	-	-	-	-	-	2	-	-	-	-	-	-	-	-	-
25 *Lasioglossum corvinum*	Ground	Polylectic	Solitary	Small	-	1	9	-	25	1	-	-	-	-	-	-	-	-	-	11	2	-	-	-	-	22	-	-	-	1	-	-	-	-	-
26 *Lasioglossum crassepunctatum*	Ground	Polylectic	Solitary	Small	-	12	1	1	-	-	1	-	-	-	-	-	1	-	-	-	1	-	-	-	1	-	-	-	-	9	3	-	-	-	-
27 *Lasioglossum discum*	Ground	Polylectic	Solitary	Medium	-	-	31	1	8	13	1	-	-	7	-	-	1	-	-	46	-	-	-	-	-	-	-	-	-	-	-	6	-	8	-
28 *Lasioglossum glabriusculum*	Ground	Polylectic	Social	Small	3	4	5	18	1	-	-	1	-	2	3	-	5	1	-	2	2	-	13	-	-	-	-	-	-	4	-	1	-	2	1
29 *Lasioglossum griseolum*	Ground	Polylectic	Solitary	Small	-	-	-	-	-	-	-	-	1	-	-	-	-	-	-	-	-	-	-	-	-	-	-	-	-	-	-	-	1	-	-
30 *Lasioglossum interruptum*	Ground	Polylectic	Social	Small	-	-	1	-	-	-	1	-	-	-	-	1	-	-	-	1	-	-	-	-	-	-	-	-	-	-	-	-	-	-	-
31 *Lasioglossum leucozonium*	Ground	Polylectic	Solitary	Medium	1	-	2	2	1	-	-	1	-	-	-	-	2	-	-	3	-	-	-	-	-	-	-	-	2	-	-	-	-	-	-
32 *Lasioglossum lineare* ˣ ▪	Ground	Polylectic	Social	Small	-	-	-	-	1	-	-	-	-	-	-	-	-	-	-	-	-	-	-	-	-	1	-	-	-	-	-	-	-	-	-
33 *Lasioglossum lucidulum*	Ground	Polylectic	Solitary	Small	-	-	-	1	-	2	-	-	-	-	-	-	1	-	-	-	-	-	-	-	-	-	-	-	2	-	-	-	-	-	-
34 *Lasioglossum malachurum*	Ground	Polylectic	Social	Medium	1	13	9	4	7	-	-	-	-	-	1	-	4	-	-	6	10	-	-	-	-	-	-	-	-	9	4	-	-	-	-
35 *Lasioglossum mesosclerum*	Ground	Polylectic	Solitary	Small	-	1	-	-	-	-	-	-	-	-	-	-	-	-	-	-	-	-	-	-	-	-	-	-	-	1	-	-	-	-	-
36 *Lasioglossum minutissimum* ˣ ▪	Ground	Polylectic	Solitary	Small	-	1	-	-	-	-	-	-	-	-	-	-	-	-	-	-	-	-	-	-	-	-	-	-	-	1	-	-	-	-	-
37 *Lasioglossum nigripes*	Ground	Polylectic	Social	Medium	-	-	1	-	-	-	-	-	-	-	-	-	-	-	-	1	-	-	-	-	-	-	-	-	-	-	-	-	-	-	-
38 *Lasioglossum pauxillum*	Ground	Polylectic	Social	Small	-	-	1	3	-	-	1	-	-	-	-	-	3	-	-	1	-	-	-	-	-	-	-	-	1	-	-	-	-	-	-
39 *Lasioglossum punctatissimum*	Ground	Polylectic	Solitary	Small	3	-	-	-	-	-	-	-	-	-	-	-	-	-	-	-	-	-	-	-	-	-	2	-	-	-	-	-	1	-	-
40 *Lasioglossum truncaticolle*	Ground	Oligolectic	Solitary	Small	-	-	13	-	22	4	-	-	-	-	-	-	-	-	-	39	-	-	-	-	-	-	-	-	-	-	-	-	-	-	-
41 *Seladonia pollinosa*	Ground	Polylectic	NA	Medium	-	-	-	-	-	-	20	-	-	-	-	3	-	-	12	3	-	-	-	-	2	-	-	-	-	-	-	-	-	-	-
42 *Seladonia seladonia*	Ground	Polylectic	Social	Small	1	15	-	5	-	-	-	-	-	-	14	-	5	-	-	-	-	-	-	-	-	-	-	-	1	1	-	-	-	-	-
43 *Seladonia subaurata*	Ground	Polylectic	Social	Small	1	1	-	6	-	5	1	-	-	1	-	-	6	-	-	-	-	-	-	-	1	-	-	-	6	1	-	-	1	-	-
***44** *Seladonia submediterranea*	Ground	Polylectic	Social	Small	1	-	1	-	-	1	-	-	-	-	1	-	-	-	-	-	-	-	-	-	-	-	-	-	1	-	-	-	-	1	-
45 *Seladonia vestita*	Ground	Polylectic	NA	Small	-	-	-	-	-	-	3	-	-	-	-	-	-	-	-	1	-	-	-	-	1	-	-	-	-	-	-	-	1	-	-
46 *Sphecodes alternatus* ˣ	Parasitic ground	NA parasitic	NA parasitic	Medium	1	-	-	-	-	-	1	-	-	-	-	-	-	-	-	-	-	-	-	-	-	-	-	-	-	-	-	-	2	-	-
47 *Sphecodes gibbus* ˣ	Parasitic ground	NA parasitic	NA parasitic	Medium	-	-	-	-	-	-	1	-	-	-	-	-	-	-	-	-	-	-	-	-	-	-	-	-	-	-	-	-	1	-	-
48 *Sphecodes monilicornis* ˣ	Parasitic ground	NA parasitic	NA parasitic	Medium	-	1	-	-	-	-	-	-	-	-	1	-	-	-	-	-	-	-	-	-	-	-	-	-	-	-	-	-	-	-	-
49 *Sphecodes reticulatus* ˣ	Parasitic ground	NA parasitic	NA parasitic	Medium	-	1	-	-	-	-	-	-	-	-	-	-	-	-	-	-	-	-	-	-	-	-	-	-	1	-	-	-	-	-	-
50 *Sphecodes rufiventris* ˣ ▪	Parasitic ground	NA parasitic	NA parasitic	Medium	-	-	-	-	-	-	1	-	-	-	-	-	-	-	-	-	-	-	-	-	-	-	-	-	-	-	-	-	1	-	-

Nomiinae																																			
51 *Nomiapis bispinosa*	Ground	Polylectic	Solitary	Medium	-	-	-	-	-	1	1	-	-	-	-	-	-	-	1	1	-	-	-	-	-	-	-	-	-	-	-	-	-	-	-
52 *Nomiapis diversipes*	Ground	Polylectic	Solitary	Medium	2	3	1	-	-	7	19	7	28	-	-	-	-	1	1	-	-	2	-	-	2	-	-	9	48	-	-	-	4	-	-
Rophitinae																																			
53 *Rophitoides canus*	Ground	Oligolectic	Solitary	Small	-	-	-	-	-	-	-	-	2	-	-	-	-	-	-	-	-	-	-	-	-	-	-	2	-	-	-	-	-	-	-
54 *Systropha curvicornis*	Ground	Oligolectic	Solitary	Medium	-	-	-	-	29	-	1	-	-	2	-	-	-	-	1	3	27	-	-	-	-	-	1	-	-	-	-	-	-	-	-
55 *Systropha planidens*	Ground	Oligolectic	Solitary	Medium	-	-	4	-	5	-	-	-	-	-	-	-	-	-	-	-	9	-	-	-	-	-	-	-	-	-	-	-	-	-	-
Melittidae																																			
Melittinae																																			
56 *Macropis frivaldszkyi*	Ground	Oligolectic	Solitary	Medium	-	-	1	-	-	-	-	-	-	-	-	-	-	-	-	-	-	-	-	-	-	-	-	-	1	-	-	-	-	-	-
Megachilidae																																			
Megachilinae																																			
57 *Anthidium florentinum* × ▪	Cavity	Polylectic	Solitary	Large	-	-	-	-	1	-	-	-	-	-	-	-	-	-	-	-	-	-	-	-	-	-	-	-	-	-	-	-	-	-	1
58 *Anthidium manicatum*	Cavity	Polylectic	Solitary	Large	-	-	-	-	-	1	-	2	-	-	-	-	-	-	-	-	-	2	-	-	-	-	-	-	1	-	-	-	-	-	-
59 *Anthidium oblongatum*	Cavity	Polylectic	Solitary	Medium	-	-	-	-	1	-	-	-	-	-	-	-	-	-	-	-	-	-	-	1	-	-	-	-	-	-	-	-	-	-	-
60 *Coelioxys inermis* ×	Parasitic cavity	NA parasitic	NA parasitic	Medium	-	-	-	-	-	-	-	1	-	-	-	-	-	-	-	-	-	-	-	-	-	-	-	-	1	-	-	-	-	-	-
61 *Heriades rubicola*	Cavity	Oligolectic	Solitary	Small	-	-	-	4	-	-	-	-	-	-	-	-	4	-	-	-	-	-	-	-	-	-	-	-	-	-	-	-	-	-	-
62 *Megachile argentata*	Cavity	Polylectic	Solitary	Medium	-	1	-	-	1	-	2	-	1	1	-	-	-	-	-	-	-	1	-	-	1	-	-	-	1	1	-	1	-	-	1
63 *Megachile centuncularis*	Cavity	Polylectic	Solitary	Medium	2	-	-	-	-	-	-	-	-	-	-	-	-	-	-	-	-	-	-	-	-	-	2	-	-	-	-	-	-	-	-
64 *Megachile deceptoria*	Cavity	Polylectic	Solitary	Large	-	-	-	-	-	-	1	-	-	-	-	-	-	-	-	-	-	1	-	-	-	-	-	-	-	-	-	-	-	-	-
65 *Megachile ericetorum*	Cavity	Oligolectic	Solitary	Large	5	-	-	-	7	-	1	-	-	-	-	-	-	-	-	-	-	1	-	-	-	-	11	-	-	-	-	1	-	-	-
66 *Megachile versicolor*	Cavity	Polylectic	Solitary	Medium	1	-	-	-	-	-	-	-	-	-	-	-	-	-	-	-	-	-	-	-	-	-	1	-	-	-	-	-	-	-	-
67 *Megachile willughbiella*	Cavity	Polylectic	Solitary	Large	3	-	-	-	-	-	-	-	-	-	-	-	-	-	-	-	-	-	-	-	-	-	3	-	-	-	-	-	-	-	-
68 *Osmia aurulenta*	Cavity	Polylectic	Solitary	Medium	-	-	1	-	-	1	-	-	-	-	-	-	-	-	-	1	-	-	-	-	-	-	-	-	1	-	-	-	-	-	-
69 *Pseudoanthidium tenellum*	Cavity	Oligolectic	Solitary	Medium	-	-	-	-	-	1	-	-	-	-	-	-	-	-	-	1	-	-	-	-	-	-	-	-	-	-	-	-	-	-	-
Apidae																																			
Anthophorinae																																			
***70** *Anthophora* cf. *crinipes*	Ground	Polylectic	Solitary	Large	-	-	-	3	-	-	-	-	-	-	-	-	-	-	-	-	-	-	3	-	-	-	-	-	-	-	-	-	-	-	-
Apinae																																			
71 *Apis mellifera*	Cavity	Polylectic	Social	Large	47	19	199	4	208	92	51	28	19	40	-	15	1	10	-	280	2	2	3	28	54	42	38	-	41	15	1	16	14	127	18
72 *Bombus argillaceus*	Ground	Polylectic	Social	Large	-	-	-	2	-	-	-	-	-	-	-	-	-	-	-	-	-	-	2	-	-	-	-	-	-	-	-	-	-	-	-
73 *Bombus hortorum*	Ground	Polylectic	Social	Large	-	-	-	3	-	-	-	-	-	-	-	-	-	-	-	-	-	-	3	-	-	-	-	-	-	-	-	-	-	-	-
***74** *Bombus terrestris* agg.	Ground	Polylectic	Social	Large	-	-	2	12	5	1	-	-	-	-	-	-	1	-	-	4	-	-	11	1	-	-	-	-	-	-	-	-	-	-	3
Eucerinae																																			
***75** *Eucera chrysopyga*	Ground	Oligolectic	Solitary	Large	14	-	-	-	1	1	-	18	49	-	-	-	-	66	-	-	-	-	-	-	-	-	14	1	1	-	-	1	-	-	-
76 *Eucera cineraria*	Ground	Oligolectic	Solitary	Medium	-	-	-	-	-	-	1	1	8	-	-	1	-	8	-	-	-	1	-	-	-	-	-	-	-	-	-	-	-	-	-
77 *Eucera clypeata*	Ground	Polylectic	Solitary	Medium	12	-	5	-	-	1	1	-	2	-	-	1	-	2	-	1	-	-	-	-	-	-	-	-	-	-	-	12	-	5	-
78 *Eucera hungarica*	Ground	Oligolectic	Solitary	Large	-	-	-	-	-	-	1	6	1	-	-	-	-	7	-	-	-	1	-	-	-	-	-	-	-	-	-	-	-	-	-
79 *Eucera interrupta*	Ground	Oligolectic	Solitary	Large	1	-	-	-	3	-	-	6	12	-	-	-	-	18	-	-	-	-	-	-	-	-	1	-	-	-	-	-	-	-	3
80 *Eucera nigrescens*	Ground	Oligolectic	Solitary	Large	-	-	-	1	-	-	-	-	-	-	-	-	-	-	-	-	-	-	1	-	-	-	-	-	-	-	-	-	-	-	-
81 *Eucera nigrifacies*	Ground	Oligolectic	Solitary	Medium	-	1	15	-	1	1	-	-	-	-	-	-	-	-	-	15	-	-	-	-	-	-	-	-	-	-	1	-	-	2	-
82 *Eucera pannonica*	Ground	Oligolectic	Solitary	Large	-	-	1	-	-	-	1	-	-	-	-	-	-	-	-	2	-	-	-	-	-	-	-	-	-	-	-	-	-	-	-
83 *Eucera proxima*	Ground	Polylectic	Solitary	Large	-	-	-	-	-	-	-	1	1	-	-	-	-	2	-	-	-	-	-	-	-	-	-	-	-	-	-	-	-	-	-
84 *Eucera punctulata* × ▪	Ground	Polylectic	Solitary	Large	-	-	-	-	-	-	-	1	-	-	-	-	-	1	-	-	-	-	-	-	-	-	-	-	-	-	-	-	-	-	-
85 *Eucera taurica* × ▪	Ground	Oligolectic	Solitary	Large	-	-	-	-	-	3	-	-	-	-	-	-	-	-	-	3	-	-	-	-	-	-	-	-	-	-	-	-	-	-	-
Nomadinae																																			
86 *Biastes brevicornis* × ▪	Parasitic ground	NA parasitic	NA parasitic	Small	-	-	-	-	1	-	-	-	-	-	-	-	-	-	-	-	1	-	-	-	-	-	-	-	-	-	-	-	-	-	-
87 *Nomada basalis* ×	Parasitic ground	NA parasitic	NA parasitic	Medium	-	-	1	-	-	-	-	-	-	-	-	-	-	-	-	-	-	-	-	-	-	-	-	-	1	-	-	-	-	-	-
88 *Nomada fucata* ×	Parasitic ground	NA parasitic	NA parasitic	Medium	1	-	1	-	-	-	1	-	-	-	-	-	-	-	-	-	-	-	-	-	1	-	-	-	2	-	-	-	-	-	-
89 *Nomada nobilis* ×	Parasitic ground	NA parasitic	NA parasitic	Large	1	-	-	-	-	-	-	-	-	-	-	-	-	-	-	-	-	-	-	-	-	-	-	-	-	-	-	1	-	-	-
90 *Nomada pectoralis* ×	Parasitic ground	NA parasitic	NA parasitic	Large	1	-	-	-	1	-	-	-	-	-	-	-	-	-	-	1	-	-	-	-	-	-	1	-	-	-	-	-	-	-	-
91 *Pasites maculatus* ×	Parasitic ground	NA parasitic	NA parasitic	Small	-	-	-	-	-	-	1	-	-	-	-	-	-	-	-	-	-	-	-	-	-	-	-	-	-	-	-	-	1	-	-
Xylocopinae																																			
92 *Ceratina acuta* × ▪	Cavity	Polylectic	Solitary	Small	-	-	-	-	-	-	1	-	-	-	-	-	-	-	-	1	-	-	-	-	-	-	-	-	-	-	-	-	-	-	-
93 *Ceratina chalcites*	Cavity	Polylectic	Solitary	Large	-	-	-	-	-	-	2	-	-	-	-	-	-	-	1	1	-	-	-	-	-	-	-	-	-	-	-	-	-	-	-
94 *Ceratina cyanea*	Cavity	Polylectic	Solitary	Small	-	1	1	-	-	-	-	-	-	-	-	-	-	-	-	1	-	-	-	-	-	-	-	-	-	1	-	-	-	-	-
95 *Ceratina nigrolabiata*	Cavity	Polylectic	Solitary	Small	-	1	1	-	-	-	1	-	-	-	-	-	-	-	-	-	-	-	-	-	-	-	-	-	2	1	-	-	-	-	-
96 *Xylocopa violacea*	Cavity	Polylectic	Solitary	Large	-	-	-	1	-	-	-	-	-	-	-	-	-	-	-	-	-	-	1	-	-	-	-	-	-	-	-	-	-	-	-
# Species					33	28	32	24	25	24	30	16	14	15	14	7	19	12	5	31	11	8	8	3	8	6	13	3	28	19	7	14	20	9	7
# Individuals					279	184	376	124	355	201	177	160	139	93	72	25	87	118	16	477	57	11	37	30	63	78	81	12	535	69	16	57	68	151	28

Note 1: × indicates species excluded from functional traits analyses
Note 2: ▪ indicates species with only male specimens collected
Note 3: * indicates taxonomic issues, commented below in the Remarks

Remarks on taxonomic issues concerning selected taxa in the dataset (identity, recognition, faunistics):

***8** ***Hylaeus* (*Lambdopsis*) sp.:** Of the two specimens in the subgenus *Lambdopsis*, one remained unidentified, and we treated it as different from *H. dilatatus*. In fact, specimen represents an aberrant individual, showing unusual mixture of characters of several species in the subgenus.

***9** ***Andrena* cf. *afzeliella*:** Taxonomy of the subgenus *Taeniandrena* was recently clarified (Praz et al. 2022), but the members of this group remain among the most challenging for identification, particularly the females in worn state of the body pubescence (practically all of female specimens in our sample, totalling 24, are in relatively poor condition). The identification of males (9) is somewhat more reliable, at least regarding the taxa considered present in the area (SE Europe), hence we believe that all *Taeniandrena* specimens in our samples belong to this single species. Anyway, we retain the level of reservation to this taxonomic outcome, in order to emphasize also the relevance of this finding for to the faunistic evidence of this species group in Serbia: this appears to be the first nominal country record of *Andrena afzeliella* (cf. Reverté et al. 2023; Sentil et al. 2026). In fact, the taxon was previously hidden under the widely misinterpreted, closely related species *Andrena ovatula* (commonly reported throughout Europe, incl. Serbia), which is now considered likely absent from SE Europe (Praz et al. 2022); therefore, most records of *ovatula* might be tentatively reinterpreted as *afzeliella*. However, few other species, poorly known and probably much less common than *afzeliella*, might be also present in the area (e.g. *A. ovata*); hence, the identity of our entire sample must remain tentative. Ecologically, all these closely related species seem to share very similar biological traits (e.g. preference for Fabaceae).

***18-19** ***Halictus brunnescens / quadricinctus*:** We identified both of these closely related species of the subgenus *H.* (*Halictus*) in our samples, all females, whose separation is based only on characters of metasomal pubescence pattern (Pesenko 2005; Pauly et al. 2016). However, these characters may sometimes show nearly overlapping variability, and moreover, it becomes exceptionally difficult to use them as differential characters when specimens are in worn condition (cf. Ebmer 1988, 2005). Since this is the case with nearly half of our specimens, we decided to analyse them as a single "morpho-taxon", while retaining the information that both species are effectively documented as present (by numbering this entry as two species).

***23** ***Halictus* (*Monilapis*) spp.:** Some of the commonest and widespread species in this subgenus are impossible to separate in the female sex – *eurygnathus*, *langobardicus* and *simplex* (Amiet et al. 2001; Pauly 2015; Pesenko et al. 2000), and all our specimens are females. Therefore, all sampled specimens in this species aggregate were treated as a single "morpho-taxon" at the subgenus level, acknowledging that it most likely includes more than one species.

***44** ***Seladonia submediterranea*:** After revision of the widely distributed species complex *S. smaragdula* s.l. (Pauly et al. 2015), distribution of the nominal species in a strict sense is restricted mostly to the Mediterranean zone, while the only species present in the more continental areas of Europe is the newly described *S. submediterranea*. However, this change was not fully reflected in most recent country-level checklist for Europe (Reverté et al. 2023; Sentil et al. 2026), neither recognised in the updated national list for Serbia (Mudri-Stojnić et al. 2021). Accordingly, our findings formally represent the first country record of this species, even though it was essentially documented long ago, under the previous species concept of *S. smaragdula*.

***70** ***Anthophora* cf. *crinipes*:** Only three female specimens of the genus *Anthophora* were collected, from the single location and the single plant, all being in a very poor condition regarding their pubescence coat. This makes them technically difficult to identify reliably. Nevertheless, the presented identity is quite plausible based on the phenology: *A. crinipes* is one of the few small *Anthophora* species in the study region, but the only one with an early-spring activity, which corresponds with the rather worn condition of the rare individuals surviving up to the early summer.

***74** ***Bombus terrestris* agg.:** Worker caste in this species complex are known as notoriously difficult to separate and identify reliably based on morphology, and most specimens in our sample were workers (19, out of 20). In the study area, only two species of this aggregate are present, i.e. *lucorum* and *terrestris*, and probably most of the specimens (if not all) belong to the later species (based on authors' wider faunistic evidence from throughout Serbia - unpublished).

***75** ***Eucera chrysopyga*:** The nomenclature of this species varied over previous decades, due to the alternating generic/subgeneric classification of the group, while taxonomic identity of the species and recognition of specimens was never problematic. In many relevant works (e.g. Amiet et al. 2007; Ghisbain et al. 2025; Scheuchl 2000) it was

recognised under the priority name *Eucera pollinosa* Smith, 1854, in accordance with nomenclatural rules (ICZN). However, that name becomes invalidated as a junior homonym of *Tetralonia pollinosa* (Lepeletier, 1841) in taxonomic arrangements where the later genus has been included in *Eucera* s.l. (e.g. Aubert M. 2020; Dorchin et al. 2018; Scheuchl & Willner 2016). To avoid possible confusions and uncertainty, particularly for the readers outside the taxonomy-related topics, we opt for this unambiguous name, even though the alternative nomenclatural variant temporarily prevails in the literature.

CITED REFERENCES:

Amiet F, Herrmann M, Müller A, & Neumeyer R. **2001**. Apidae 3 – *Halictus*, *Lasioglossum*. Fauna Helvetica 6.: Schweizer Zentrum für die Kartografie der Fauna & Schweizerische Entomologische Gesellschaft, Neuchatel.

Amiet F, Herrmann M, Müller A, & Neumeyer R. **2007**. Apidae 5 – *Ammobates*, *Ammobatoides*, *Anthophora*, *Biastes*, *Ceratina*, *Dasypoda*, *Epeoloides*, *Epeolus*, *Eucera*, *Macropis*, *Melecta*, *Melitta*, *Nomada*, *Pasites*, *Tetralonia*, *Thyreus*, *Xylocopa*. Fauna Helvetica 20. Schweizer Zentrum für die Kartografie der Fauna & Schweizerische Entomologische Gesellschaft, Neuchatel.

Aubert M. **2020**. Proposition de clé d'identification des Eucerini (Hymenoptera : Anthophila) de France continentale. Version provisoire. Observatoire des Abeilles. https://oabeilles.net/projets/eucerini

Ebmer AW. **1988**. Kritische Liste der nicht-parasitischen Halictidae Österreichs mit Berücksichtigung aller mitteleuropäischen Arten (Insecta: Hymenoptera: Apoidea: Halictidae). Linzer biologische Beiträge 20(2): 527–711.

Ebmer AW. **2005**. Zur Bienenfauna der Mongolei Die Arten der Gattungen *Halictus* Latr. und *Lasioglossum* Curt. (Insecta: Hymenoptera: Apoidea: Halictidae: Halictinae) Ergänzungen und Korrekturen. Linzer biologische Beiträge 37(1): 343–392.

Ghisbain G, Rosa P, Bogusch P, Brau T, Carion F, Manincor ND, et al. **2025**. The new annotated checklist of the wild bees of Europe (Hymenoptera: Anthophila)—version 2025. Zootaxa 5736(1): 1-126. doi: 10.11646/zootaxa.5736.1.1.

Mudri-Stojnić S, Andrić A, Markov-Ristić Z, Đukić A, & Vujić A. **2021**. Contribution to the knowledge of the bee fauna (Hymenoptera, Apoidea, Anthophila) in Serbia. ZooKeys 1053: 43–105. doi: 10.3897/zookeys.1053.67288.

Pauly A. **2015**. Clés illustrées pour l'identification des abeilles de Belgique et des régions limitrophes (Hymenoptera: Apoidea). I. Halictidae. Document de Travail du Projet BELBEES.

Pauly A, Devalez J, Sonet G, Nagy ZT, & Boeve J-L. **2015**. DNA barcoding and male genital morphology reveal five new cryptic species in the West Palearctic bee *Seladonia smaragdula* (Vachal, 1895) (Hymenoptera: Apoidea: Halictidae). Zootaxa 4034(2): 257–290. doi: 10.11646/zootaxa.4034.2.2.

Pauly A, Pesenko YA, & Radchenko VG. **2016**. Les *Halictus* Latreille, 1804 d'Europe et du Bassin Méditerranéen. Atlas Hymenoptera. Mons, Gembloux [visited Feb 2017]. Available from http://www.atlashymenoptera.net/page.asp?id=70.

Pesenko YA, Banaszak J, Radchenko VG, & Cierzniak T. **2000**. Bees of the family Halictidae (excluding *Sphecodes*) of Poland: taxonomy, ecology, bionomics. Wydawnictwo Uczelniane Wyzszej Szkofy Pedagogicznej, Bydgoszcz.

Pesenko YA. **2005**. Contributions to the halictid fauna of the Eastern Palaearctic Region: genus *Halictus* Latreille (Hymenoptera: Halictidae, Halictinae). Far Eastern Entomologist 150: 1–24.

Praz CJ, Genoud D, Vaucher K, Bénon D, Monks J, & Wood TJ. **2022**. Unexpected levels of cryptic diversity in European bees of the genus *Andrena* subgenus *Taeniandrena* (Hymenoptera, Andrenidae): implications for conservation. Journal of Hymenoptera Research 91: 375–428. doi: 10.3897/jhr.91.82761.

Reverté S, Miličić M, Ačanski J, Andrić A, Aracil A, Aubert M, et al. **2023**. National records of 3000 European bee and hoverfly species: A contribution to pollinator conservation. Insect Conservation and Diversity 16(6): 758-775. doi: 10.1111/icad.12680.

Scheuchl E. **2000**. Illustrierte Bestimmungstabellen der Wildbienen Deutschlands und Österreichs. Band I: Schlüssel der Gattungen und der Arten der Familie Anthophoridae. 2. erweiterte Auflage. Edited by Scheuchl E: Velden (Selbstverlag).

Scheuchl E, & Willner W. **2016**. Taschenlexikon der Wildbienen Mitteleuropas: Alle Arten im Porträt Gebundene Ausgabe. Quelle & Meyer.

Sentil A, Miličić M, Benrezkallah J, Ačanski J, Andrić A, Aubert M, et al. **2026**. Synthesised database of wild bee and hoverfly records in Europe. Scientific Data 13(1): 227. doi: 10.1038/s41597-026-06644-2.

Table S2. Geographical coordinates of the ten study sites and their landscape composition across three spatial scales.

Scale: 500 m

Site	Latitude (°)	Longitude (°)	Arable land (%)	Semi-natural habitats (%)	Rural settlements (%)	Roads (%)	Water bodies (%)
L1	45.180704	20.497835	47.64	46.02	0.38	2.15	3.81
L2	45.119782	20.393423	7.24	78.44	6.74	1.79	5.79
L3	45.115473	20.469605	80.01	19.07	0.00	0.92	0.00
L4	45.041789	20.449097	61.23	19.53	16.01	3.23	0.00
L5	44.953899	20.567986	68.13	29.08	0.72	2.07	0.00
L6	45.006007	20.617445	93.21	4.77	0.14	1.88	0.00
L7	45.117744	20.688882	90.29	7.92	0.00	1.79	0.00
L8	45.147840	20.743026	94.63	3.98	0.00	1.39	0.00
L9	45.176533	20.758870	89.74	8.51	0.00	1.75	0.00
L10	45.223413	20.798607	65.65	27.85	4.26	2.24	0.00

Scale: 1000 m

Site	Latitude (°)	Longitude (°)	Arable land (%)	Semi-natural habitats (%)	Rural settlements (%)	Roads (%)	Water bodies (%)
L1	45.180704	20.497835	51.58	43.56	0.69	1.83	2.34
L2	45.119782	20.393423	19.10	55.48	19.44	2.05	3.93
L3	45.115473	20.469605	73.56	18.14	0.00	1.09	7.21
L4	45.041789	20.449097	61.71	18.62	16.45	2.91	0.31
L5	44.953899	20.567986	80.61	14.32	1.89	2.89	0.29
L6	45.006007	20.617445	78.56	8.55	10.76	2.13	0.00
L7	45.117744	20.688882	95.28	3.13	0.00	1.59	0.00
L8	45.147840	20.743026	96.65	2.14	0.00	1.21	0.00
L9	45.176533	20.758870	94.60	4.06	0.00	1.34	0.00
L10	45.223413	20.798607	80.94	16.20	1.07	1.79	0.00

Scale: 1500 m

Site	Latitude (°)	Longitude (°)	Arable land (%)	Semi-natural habitats (%)	Rural settlements (%)	Roads (%)	Water bodies (%)
L1	45.180704	20.497835	44.53	45.68	5.49	1.55	2.75
L2	45.119782	20.393423	32.33	45.61	17.30	2.03	2.73
L3	45.115473	20.469605	64.88	24.09	0.00	1.61	9.42
L4	45.041789	20.449097	55.56	27.42	14.05	2.47	0.50
L5	44.953899	20.567986	59.06	30.48	5.42	2.37	2.67
L6	45.006007	20.617445	73.62	4.94	19.37	2.07	0.00
L7	45.117744	20.688882	96.32	2.26	0.00	1.42	0.00
L8	45.147840	20.743026	96.87	1.85	0.00	1.28	0.00
L9	45.176533	20.758870	96.01	2.70	0.00	1.29	0.00
L10	45.223413	20.798607	87.95	10.15	0.48	1.42	0.00

Table S3. Compiled iNEXT output of observed characteristics and estimated diversity metrics for the full dataset, standardized to a fixed sample coverage of 89.7% (estimateD function).

Site	Observed species	Observed individuals	Observed coverage	Targeted coverage	Method	Subsample size (m)	Species richness (q = 0) qD [LCL, UCL]	Shannon diveristy (q = 1) qD [LCL, UCL]	Simpson diversity (q = 2) qD [LCL, UCL]
L1	33	279	0.943	0.897	Rarefaction	87.81	18.84 [11.86, 25.82]	9.08 [7.31, 10.85]	5.47 [4.51, 6.42]
L2	28	184	0.919	0.897	Rarefaction	68.17	17.98 [3.20, 32.77]	12.24 [8.90, 15.58]	9.25 [7.31, 11.18]
L3	32	376	0.958	0.897	Rarefaction	67.48	14.36 [10.08, 18.64]	5.83 [4.69, 6.96]	3.19 [2.68, 3.71]
L4	24	124	0.944	0.897	Rarefaction	69.20	19.97 [13.44, 26.51]	13.70 [10.64, 16.75]	10.25 [7.95, 12.55]
L5	25	355	0.969	0.897	Rarefaction	48.67	10.72 [8.13, 13.31]	4.60 [3.81, 5.39]	2.67 [2.30, 3.04]
L6	24	201	0.930	0.897	Rarefaction	70.48	13.63 [0.00, 28.95]	5.51 [3.78, 7.25]	3.46 [2.84, 4.08]
L7	30	177	0.876	0.897	Extrapolation	354.00	50.10 [0.00, 128.03]	9.05 [4.98, 13.11]	4.92 [4.03, 5.81]
L8	16	160	0.963	0.897	Rarefaction	37.47	9.13 [6.56, 11.70]	5.22 [4.13, 6.31]	3.57 [2.85, 4.28]
L9	14	139	0.964	0.897	Rarefaction	25.69	7.58 [5.69, 9.47]	5.53 [4.50, 6.56]	4.38 [3.57, 5.20]
L10	14	55	0.910	0.897	Rarefaction	45.81	13.11 [5.22, 21.00]	9.43 [6.22, 12.65]	7.40 [5.02, 9.78]

Note: Values in columns q = 0, q = 1, and q = 2 represent diversity estimates with 95% confidence intervals in brackets [Lower Confidence Level, Upper Confidence Level]. Lower confidence intervals (LCL) of 0.00 for q = 0 are artifactual results of the asymptotic normal approximation used by iNEXT when dealing with high numbers of rare species (singletons).

Table S4. Compiled iNEXT output of observed characteristics and estimated diversity metrics for the dataset with *Apis mellifera* excluded, standardized to a fixed sample coverage of 85.5% (estimateD function).

Site	Observed species	Observed individuals	Observed coverage	Targeted coverage	Method	Subsample size (m)	Species richness (q = 0) qD [LCL, UCL]	Shannon diveristy (q = 1) qD [LCL, UCL]	Simpson diversity (q = 2) qD [LCL, UCL]
L1	32	232	0.931	0.855	Rarefaction	53.51	15.22 [10.05, 20.40]	7.77 [5.92, 9.62]	4.43 [3.44, 5.42]
L2	27	165	0.909	0.855	Rarefaction	42.63	14.60 [9.33, 19.88]	10.47 [8.11, 12.83]	7.91 [6.31, 9.51]
L3	31	177	0.910	0.855	Rarefaction	60.72	18.48 [9.85, 27.11]	11.74 [8.67, 14.81]	8.29 [6.49, 10.09]
L4	23	120	0.942	0.855	Rarefaction	48.35	16.70 [11.93, 21.47]	12.04 [9.28, 14.79]	9.25 [7.18, 11.33]
L5	24	147	0.925	0.855	Rarefaction	43.45	14.12 [9.32, 18.91]	9.92 [7.61, 12.24]	7.70 [6.16, 9.24]
L6	23	109	0.872	0.855	Rarefaction	66.22	17.24 [0.00, 52.80]	7.47 [2.52, 12.43]	3.97 [2.57, 5.37]
L7	29	126	0.826	0.855	Extrapolation	252.00	49.09 [0.00, 121.37]	9.46 [3.99, 14.92]	4.19 [3.13, 5.26]
L8	15	132	0.955	0.855	Rarefaction	25.28	7.37 [5.13, 9.61]	4.11 [3.11, 5.12]	2.73 [2.13, 3.34]
L9	13	120	0.959	0.855	Rarefaction	16.57	5.86 [4.34, 7.37]	4.37 [3.47, 5.28]	3.49 [2.82, 4.17]
L10	13	47	0.895	0.855	Rarefaction	31.23	11.05 [5.38, 16.72]	8.09 [5.14, 11.04]	6.21 [3.94, 8.48]

Note: Values in columns q = 0, q = 1, and q = 2 represent diversity estimates with 95% confidence intervals in brackets [Lower Confidence Level, Upper Confidence Level]. Lower confidence intervals (LCL) of 0.00 for q = 0 are artifactual results of the asymptotic normal approximation used by iNEXT when dealing with high numbers of rare species (singletons).

Table S5. Results of Moran's I test for spatial autocorrelation across three spatial scales.

Scale: 500m

Model	Moran's I	p-value
**Total: all bees**	-0.272	0.095
**Total: wild bees**	-0.159	0.630
**Females: all bees**	-0.235	0.185
**Females: wild bees**	-0.061	0.611
**Males: wild bees**	-0.175	0.516
Apis mellifera	-0.117	0.950
**Females: *Andrena flavipes***	-0.199	0.377
**Females: oligolectic**	-0.115	0.969
**Females: polylectic**	-0.115	0.965
**Females: solitary**	-0.208	0.337
**Females: social**	-0.159	0.608
**Females: cavity nesting**	-0.065	0.632
**Females: ground nesting**	-0.067	0.647
**Females: small**	-0.086	0.802
**Females: medium**	-0.111	0.996
**Females: large**	-0.144	0.726
**Species richness: all bees**	-0.025	0.357
**Species richness: wild bees**	-0.009	0.271
**Shannon diversity: all bees**	-0.059	0.605
**Shannon diversity: wild bees**	-0.134	0.820
**Simpson diversity: all bees**	-0.126	0.881
**Simpson diversity: wild bees**	-0.121	0.920

Scale: 1000m

Model	Moran's I	p-value
**Total: all bees**	-0.156	0.649
**Total: wild bees**	-0.205	0.327
**Females: all bees**	-0.162	0.621
**Females: wild bees**	-0.060	0.611
**Males: wild bees**	-0.159	0.631
Apis mellifera	0.012	0.218
**Females: *Andrena flavipes***	-0.145	0.736
**Females: oligolectic**	-0.114	0.969
**Females: polylectic**	-0.115	0.965
**Females: solitary**	-0.233	0.219
**Females: social**	-0.184	0.429
**Females: cavity nesting**	-0.130	0.836
**Females: ground nesting**	-0.066	0.647
**Females: small**	-0.152	0.674
**Females: medium**	-0.110	0.996
**Females: large**	-0.134	0.802
**Species richness: all bees**	-0.051	0.522
**Species richness: wild bees**	-0.023	0.342
**Shannon diversity: all bees**	-0.065	0.645
**Shannon diversity: wild bees**	-0.133	0.820
**Simpson diversity: all bees**	-0.048	0.522
**Simpson diversity: wild bees**	-0.120	0.920

Scale: 1500m

Model	Moran's I	p-value
**Total: all bees**	-0.125	0.887
**Total: wild bees**	-0.231	0.227
**Females: all bees**	-0.159	0.629
**Females: wild bees**	-0.060	0.611
**Males: wild bees**	-0.081	0.764
Apis mellifera	-0.145	0.723
**Females: *Andrena flavipes***	-0.091	0.837
**Females: oligolectic**	-0.114	0.969
**Females: polylectic**	-0.115	0.965
**Females: solitary**	-0.213	0.299
**Females: social**	-0.184	0.429
**Females: cavity nesting**	-0.148	0.697
**Females: ground nesting**	-0.066	0.647
**Females: small**	-0.169	0.553
**Females: medium**	-0.110	0.996
**Females: large**	-0.134	0.802
**Species richness: all bees**	-0.072	0.672
**Species richness: wild bees**	-0.107	0.966
**Shannon diversity: all bees**	-0.049	0.538
**Shannon diversity: wild bees**	-0.133	0.820
**Simpson diversity: all bees**	-0.063	0.625
**Simpson diversity: wild bees**	-0.120	0.920

Table S6. Results of generalized linear mixed models (GLMMs) examining the effects of four landscape variables and one local variable (flowering species richness) on bee abundance across three spatial scales (A = 500 m; B = 1000 m; C = 1500 m), including total bee abundance, bee subsets, and functional group abundances.

A **Scale: 500 m**

Response variable	Statistic	Intercept	Semi-natural habitats	Rural settlements	Roads	Water bodies	Flowering species richness
	β (SE)	0.622 (0.137)		-0.049 (0.011)		0.352 (0.095)	-0.040 (0.010)
**Total: all bees**	z	4.542	dropped (n.s.)	-4.691	dropped (n.s.)	3.688	-3.849
	p	<0.001		<0.001		<0.001	<0.001
	β (SE)	-0.181 (0.100)	0.003 (0.003)				
**Total: wild bees**	z	-1.807	0.932	dropped (n.s.)	dropped (n.s.)	dropped (n.s.)	dropped (n.s.)
	p	n.s.	n.s.				
	β (SE)	0.587 (0.138)	-0.006 (0.003)	-0.044 (0.010)		0.564 (0.123)	-0.049 (0.011)
**Females: all bees**	z	4.247	-2.302	-4.183	dropped (n.s.)	4.578	-4.539
	p	<0.001	<0.05	<0.001		<0.001	<0.001
	β (SE)	-0.029 (0.179)					-0.028 (0.016)
**Females: wild bees**	z	-0.159	dropped (n.s.)	dropped (n.s.)	dropped (n.s.)	dropped (n.s.)	-1.771
	p	n.s.					n.s.
	β (SE)	0.909 (0.215)	0.013 (0.007)	-0.135 (0.062)			
**Males: wild bees**	z	4.234	1.709	-2.165	dropped (n.s.)	dropped (n.s.)	dropped (n.s.)
	p	<0.001	n.s.	<0.05			
	β (SE)	0.169 (0.403)		-0.247 (0.040)		0.909 (0.289)	-0.132 (0.032)
Apis mellifera	z	0.421	dropped (n.s.)	-6.157	dropped (n.s.)	3.146	-4.135
	p	n.s.		<0.001		<0.01	<0.001
	β (SE)	0.384 (1.466)			-1.639 (0.775)		
**Females: *Andrena flavipes***	z	0.262	dropped (n.s.)	dropped (n.s.)	-2.116	dropped (n.s.)	dropped (n.s.)
	p	n.s.			<0.05		
	β (SE)	-4.147 (0.898)				0.670 (0.599)	0.122 (0.063)
**Females: oligolectic**	z	-4.616	dropped (n.s.)	dropped (n.s.)	dropped (n.s.)	1.118	1.938
	p	<0.001				n.s.	0.053
	β (SE)	0.093 (0.262)					-0.067 (0.023)
**Females: polylectic**	z	0.355	dropped (n.s.)	dropped (n.s.)	dropped (n.s.)	dropped (n.s.)	-2.952
	p	n.s.					<0.01
	β (SE)	-0.061 (0.447)			-0.439 (0.229)		
**Females: solitary**	z	-0.136	dropped (n.s.)	dropped (n.s.)	-1.914	dropped (n.s.)	dropped (n.s.)
	p	n.s.			0.056		
	β (SE)	-1.854 (0.562)		0.084 (0.037)		1.192 (0.393)	-0.115 (0.045)
**Females: social**	z	-3.296	dropped (n.s.)	2.294	dropped (n.s.)	3.031	-2.532
	p	<0.001		<0.05		<0.01	<0.05
	β (SE)	-5.244 (0.761)			0.782 (0.345)		
**Females: cavity nesting**	z	-6.893	dropped (n.s.)	dropped (n.s.)	2.267	dropped (n.s.)	dropped (n.s.)
	p	<0.001			<0.05		
	β (SE)	-0.037 (0.201)					-0.032 (0.170)
**Females: ground nesting**	z	-0.187	dropped (n.s.)	dropped (n.s.)	dropped (n.s.)	dropped (n.s.)	-1.849
	p	n.s.					n.s.
	β (SE)	-1.874 (0.425)				1.328 (0.299)	-0.124 (0.035)
**Females: small**	z	-4.411	dropped (n.s.)	dropped (n.s.)	dropped (n.s.)	4.441	-3.542
	p	<0.001				<0.001	<0.001
	β (SE)	-0.178 (0.256)					-0.063 (0.022)
**Females: medium**	z	-0.698	dropped (n.s.)	dropped (n.s.)	dropped (n.s.)	dropped (n.s.)	-2.798
	p	n.s.					<0.01
	β (SE)	-5.909 (1.124)			0.828 (0.419)		0.173 (0.048)
**Females: large**	z	-5.259	dropped (n.s.)	dropped (n.s.)	1.937	dropped (n.s.)	3.598
	p	<0.001			<0.05		<0.001

B **Scale: 1000 m**

Response variable	Statistic	Intercept	Semi-natural habitats	Rural settlements	Roads	Water bodies	Flowering species richness
	β (SE)	0.782 (0.193)		-0.030 (0.009)		0.279 (0.110)	-0.049 (0.014)
**Total: all bees**	z	4.058	dropped (n.s.)	-3.264	dropped (n.s.)	2.530	-3.500
	p	<0.001		<0.01		<0.05	<0.001
	β (SE)	-0.222 (0.096)	0.006 (0.004)				
**Total: wild bees**	z	-2.313	1.571	dropped (n.s.)	dropped (n.s.)	dropped (n.s.)	dropped (n.s.)
	p	<0.05	n.s.				

	β (SE)	0.751 (0.201)		-0.030 (0.009)		0.321 (0.115)	-0.06 (0.015)
**Females: all bees**	z	3.744	dropped (n.s.)	-3.187	dropped (n.s.)	2.792	-4.234
	p	<0.001		<0.01		<0.01	<0.001
	β (SE)	-0.029 (0.179)					-0.028 (0.016)
**Females: wild bees**	z	-0.159	dropped (n.s.)	dropped (n.s.)	dropped (n.s.)	dropped (n.s.)	-1.771
	p	n.s.					n.s.
	β (SE)	0.876 (0.216)	0.018 (0.009)	-0.056 (0.024)			
**Males: wild bees**	z	4.056	2.105	-2.344	dropped (n.s.)	dropped (n.s.)	dropped (n.s.)
	p	<0.001	<0.05	<0.05			
	β (SE)	0.930 (0.845)		-0.117 (0.041)			-0.167 (0.064)
Apis mellifera	z	1.101	dropped (n.s.)	-2.837	dropped (n.s.)	dropped (n.s.)	-2.604
	p	n.s.		<0.01			<0.01
	β (SE)	0.463 (1.285)			-1.712 (0.701)		
**Females: *Andrena flavipes***	z	0.360	dropped (n.s.)	dropped (n.s.)	-2.444	dropped (n.s.)	dropped (n.s.)
	p	n.s.			<0.05		
	β (SE)	-4.147 (0.898)				0.670 (0.599)	0.122 (0.063)
**Females: oligolectic**	z	-4.616	dropped (n.s.)	dropped (n.s.)	dropped (n.s.)	1.118	1.938
	p	<0.001				n.s.	0.053
	β (SE)	0.093 (0.262)					-0.067 (0.023)
**Females: polylectic**	z	0.355	dropped (n.s.)	dropped (n.s.)	dropped (n.s.)	dropped (n.s.)	-2.952
	p	n.s.					<0.01
	β (SE)	0.023 (0.354)			-0.488 (0.183)		
**Females: solitary**	z	0.065	dropped (n.s.)	dropped (n.s.)	-2.671	dropped (n.s.)	dropped (n.s.)
	p	n.s.			<0.01		
	β (SE)	-1.444 (0.603)				1.567 (0.425)	-0.155 (0.048)
**Females: social**	z	-2.393	dropped (n.s.)	dropped (n.s.)	dropped (n.s.)	3.684	-3.211
	p	<0.05				<0.001	<0.01
	β (SE)	-5.056 (0.708)			0.684 (0.318)		
**Females: cavity nesting**	z	-7.143	dropped (n.s.)	dropped (n.s.)	2.152	dropped (n.s.)	dropped (n.s.)
	p	<0.001			<0.05		
	β (SE)	-0.037 (0.201)					-0.032 (0.170)
**Females: ground nesting**	z	-0.187	dropped (n.s.)	dropped (n.s.)	dropped (n.s.)	dropped (n.s.)	-1.849
	p	n.s.					n.s.
	β (SE)	-2.489 (0.464)			0.019 (0.009)	1.242 (0.255)	-0.111 (0.030)
**Females: small**	z	-5.365	dropped (n.s.)	dropped (n.s.)	2.229	4.866	-3.704
	p	<0.001			<0.05	<0.001	<0.001
	β (SE)	-0.178 (0.256)					-0.063 (0.022)
**Females: medium**	z	-0.698	dropped (n.s.)	dropped (n.s.)	dropped (n.s.)	dropped (n.s.)	-2.798
	p	n.s.					<0.01
	β (SE)	-4.155 (0.004)					0.156 (0.004)
**Females: large**	z	-985.13	dropped (n.s.)	dropped (n.s.)	dropped (n.s.)	dropped (n.s.)	37.49
	p	<0.001					<0.001

C **Scale: 1500 m**

**Response variable**	**Statistic**	**Intercept**	**Semi-natural habitats**	**Rural settlements**	**Roads**	**Water bodies**	**Flowering species richness**
	β (SE)	0.532 (0.183)					-0.027 (0.016)
**Total: all bees**	z	2.909	dropped (n.s.)	dropped (n.s.)	dropped (n.s.)	dropped (n.s.)	-1.748
	p	<0.001					n.s.
	β (SE)	0.028 (0.284)			-0.172 (0.183)	0.202 (0.165)	
**Total: wild bees**	z	0.100	dropped (n.s.)	dropped (n.s.)	-0.779	1.222	dropped (n.s.)
	p	n.s.			n.s.	n.s.	
	β (SE)	0.496 (0.191)					-0.038 (0.016)
**Females: all bees**	z	2.602	dropped (n.s.)	dropped (n.s.)	dropped (n.s.)	dropped (n.s.)	-2.369
	p	<0.001					<0.05
	β (SE)	-0.029 (0.179)					-0.028 (0.016)
**Females: wild bees**	z	-0.159	dropped (n.s.)	dropped (n.s.)	dropped (n.s.)	dropped (n.s.)	-1.771
	p	n.s.					n.s.
	β (SE)	2.329 (0.598)	0.016 (0.009)		-0.989 (0.403)		
**Males: wild bees**	z	3.892	1.756	dropped (n.s.)	-2.452	dropped (n.s.)	dropped (n.s.)
	p	<0.001	n.s.		<0.05		
	β (SE)	-0.522 (0.693)					-0.087 (0.059)
Apis mellifera	z	-0.753	dropped (n.s.)	dropped (n.s.)	dropped (n.s.)	dropped (n.s.)	-1.501
	p	n.s.					n.s.

**Females: *Andrena flavipes***	β (SE)	3.631 (2.122)	dropped (n.s.)	0.194 (0.081)	-4.365 (1.496)	dropped (n.s.)	dropped (n.s.)
	z	1.711		2.385	-2.917		
	p	n.s.		<0.05	<0.01		
**Females: oligolectic**	β (SE)	-4.147 (0.898)	dropped (n.s.)	dropped (n.s.)	dropped (n.s.)	0.670 (0.599)	0.122 (0.063)
	z	-4.616				1.118	1.938
	p	<0.001				n.s.	0.053
**Females: polylectic**	β (SE)	0.093 (0.262)	dropped (n.s.)	dropped (n.s.)	dropped (n.s.)	dropped (n.s.)	-0.067 (0.023)
	z	0.355					-2.952
	p	n.s.					<0.01
**Females: solitary**	β (SE)	0.164 (0.502)	dropped (n.s.)	dropped (n.s.)	-0.601 (0.281)	dropped (n.s.)	dropped (n.s.)
	z	0.327			-2.142		
	p	n.s.			<0.05		
**Females: social**	β (SE)	-1.444 (0.603)	dropped (n.s.)	dropped (n.s.)	dropped (n.s.)	1.567 (0.425)	-0.155 (0.048)
	z	-2.393				3.684	-3.211
	p	<0.05				<0.001	<0.01
**Females: cavity nesting**	β (SE)	-5.090 (0.966)	dropped (n.s.)	dropped (n.s.)	0.751 (0.494)	dropped (n.s.)	dropped (n.s.)
	z	-5.271			1.521		
	p	<0.001			n.s.		
**Females: ground nesting**	β (SE)	-0.037 (0.201)	dropped (n.s.)	dropped (n.s.)	dropped (n.s.)	dropped (n.s.)	-0.032 (0.170)
	z	-0.187					-1.849
	p	n.s.					n.s.
**Females: small**	β (SE)	-3.721 (0.789)	dropped (n.s.)	dropped (n.s.)	0.908 (0.337)	0.917 (0.285)	-0.078 (0.033)
	z	-4.711			2.697	3.214	-2.360
	p	<0.001			<0.01	<0.01	<0.05
**Females: medium**	β (SE)	-0.178 (0.256)	dropped (n.s.)	dropped (n.s.)	dropped (n.s.)	dropped (n.s.)	-0.063 (0.022)
	z	-0.698					-2.798
	p	n.s.					<0.01
**Females: large**	β (SE)	-4.155 (0.004)	dropped (n.s.)	dropped (n.s.)	dropped (n.s.)	dropped (n.s.)	0.156 (0.004)
	z	-985.13					37.49
	p	<0.001					<0.001

Note: β = estimated coefficient; SE = standard error; *z* = Wald *z* -value; *p* = significance level; n.s. = not significant; "dropped" indicates variables removed during model simplification due to non-significant effects.

Table S7. Results of linear regression models (LMs) examining the effects of four landscape variables and one local variable (flowering species richness) on bee diversity metrics across three spatial scales.

A **Scale: 500 m**

Response variable	Statistic	Intercept	Semi-natural habitats	Rural settlements	Roads	Water bodies	Flowering species richness
Species richness: all bees	β (SE)	2.313 (0.586)			0.208 (0.295)		
	t	3.946	dropped (n.s.)	dropped (n.s.)	0.704	dropped (n.s.)	dropped (n.s.)
	p	<0.01			n.s.		
Species richness: wild bees	β (SE)	2.429 (0.650)			0.142 (0.328)		
	t	3.736	dropped (n.s.)	dropped (n.s.)	0.432	dropped (n.s.)	dropped (n.s.)
	p	<0.01			n.s.		
Shannon diversity: all bees	β (SE)	1.822 (0.097)		0.055 (0.017)			
	t	18.843	dropped (n.s.)	3.198	dropped (n.s.)	dropped (n.s.)	dropped (n.s.)
	p	<0.001		<0.05			
Shannon diversity: wild bees	β (SE)	1.854 (0.134)				0.484 (0.190)	
	t	13.792	dropped (n.s.)	dropped (n.s.)	dropped (n.s.)	2.547	dropped (n.s.)
	p	<0.001				<0.05	
Simpson diversity: all bees	β (SE)	1.349 (0.102)		0.068 (0.018)			
	t	13.217	dropped (n.s.)	3.771	dropped (n.s.)	dropped (n.s.)	dropped (n.s.)
	p	<0.001		<0.01			
Simpson diversity: wild bees	β (SE)	1.401 (0.142)				0.589 (0.201)	
	t	9.850	dropped (n.s.)	dropped (n.s.)	dropped (n.s.)	2.929	dropped (n.s.)
	p	<0.001				<0.05	

B **Scale: 1000 m**

Response variable	Statistic	Intercept	Semi-natural habitats	Rural settlements	Roads	Water bodies	Flowering species richness
Species richness: all bees	β (SE)	2.445 (0.539)			0.142 (0.275)		
	t	4.534	dropped (n.s.)	dropped (n.s.)	0.517	dropped (n.s.)	dropped (n.s.)
	p	<0.01			n.s.		
Species richness: wild bees	β (SE)	2.206 (0.568)			0.265 (0.289)		
	t	3.886	dropped (n.s.)	dropped (n.s.)	0.916	dropped (n.s.)	dropped (n.s.)
	p	<0.01			n.s.		
Shannon diversity: all bees	β (SE)	1.809 (0.115)		0.033 (0.013)			
	t	15.711	dropped (n.s.)	2.524	dropped (n.s.)	dropped (n.s.)	dropped (n.s.)
	p	<0.001		<0.05			
Shannon diversity: wild bees	β (SE)	1.854 (0.134)				0.484 (0.190)	
	t	13.792	dropped (n.s.)	dropped (n.s.)	dropped (n.s.)	2.547	dropped (n.s.)
	p	<0.001				<0.05	
Simpson diversity: all bees	β (SE)	1.328 (0.123)		0.04 (0.014)			
	t	10.823	dropped (n.s.)	3.035	dropped (n.s.)	dropped (n.s.)	dropped (n.s.)
	p	<0.001		<0.05			
Simpson diversity: wild bees	β (SE)	1.401 (0.142)				0.589 (0.201)	
	t	9.850	dropped (n.s.)	dropped (n.s.)	dropped (n.s.)	2.929	dropped (n.s.)
	p	<0.001				<0.05	

C **Scale: 1500 m**

Response variable	Statistic	Intercept	Semi-natural habitats	Rural settlements	Roads	Water bodies	Flowering species richness
Species richness: all bees	β (SE)	2.401 (0.698)			0.178 (0.390)		
	t	3.441	dropped (n.s.)	dropped (n.s.)	0.455	dropped (n.s.)	dropped (n.s.)
	p	<0.01			n.s.		
Species richness: wild bees	β (SE)	1.992 (0.725)			0.408 (0.405)		
	t	2.748	dropped (n.s.)	dropped (n.s.)	1.006	dropped (n.s.)	dropped (n.s.)
	p	<0.05			n.s.		
Shannon diversity: all bees	β (SE)	1.844 (0.148)		0.021 (0.015)			
	t	12.430	dropped (n.s.)	1.388	dropped (n.s.)	dropped (n.s.)	dropped (n.s.)
	p	<0.001		n.s.			
Shannon diversity: wild bees	β (SE)	1.854 (0.134)				0.484 (0.190)	
	t	13.792	dropped (n.s.)	dropped (n.s.)	dropped (n.s.)	2.547	dropped (n.s.)
	p	<0.001				<0.05	

**Simpson diversity: all bees**	β (SE)	1.373 (0.169)		0.027 (0.017)			
	t	12.430	dropped (n.s.)	1.551	dropped (n.s.)	dropped (n.s.)	dropped (n.s.)
	p	<0.001		n.s.			
**Simpson diversity: wild bees**	β (SE)	1.401 (0.142)				0.589 (0.201)	
	t	9.850	dropped (n.s.)	dropped (n.s.)	dropped (n.s.)	2.929	dropped (n.s.)
	p	<0.001				<0.05	

Note: β = estimated coefficient; SE = standard error; t = Student's t-value; p = significance level; n.s. = not significant; "dropped" indicates variables removed during model simplification due to non-significant effects.
